# Integrated multi-omics and artificial intelligence to explore new neutrophils clusters and potential biomarkers in sepsis with experimental validation

**DOI:** 10.3389/fimmu.2024.1377817

**Published:** 2024-05-29

**Authors:** Peng Xu, Zuo Tao, Cheng Zhang

**Affiliations:** Department of General Surgery, General Hospital of Northern Theater Command, Shenyang, Liaoning, China

**Keywords:** sepsis, neutrophils, single cell, hdWGCNA, machine learning

## Abstract

**Background:**

Sepsis, causing serious organ and tissue damage and even death, has not been fully elucidated. Therefore, understanding the key mechanisms underlying sepsis-associated immune responses would lead to more potential therapeutic strategies.

**Methods:**

Single-cell RNA data of 4 sepsis patients and 2 healthy controls in the GSE167363 data set were studied. The pseudotemporal trajectory analyzed neutrophil clusters under sepsis. Using the hdWGCNA method, key gene modules of neutrophils were explored. Multiple machine learning methods were used to screen and validate hub genes for neutrophils. SCENIC was then used to explore transcription factors regulating hub genes. Finally, quantitative reverse transcription-polymerase chain reaction was to validate mRNA expression of hub genes in peripheral blood neutrophils of two mice sepsis models.

**Results:**

We discovered two novel neutrophil subtypes with a significant increase under sepsis. These two neutrophil subtypes were enriched in the late state during neutrophils differentiation. The hdWGCNA analysis of neutrophils unveiled that 3 distinct modules (Turquoise, brown, and blue modules) were closely correlated with two neutrophil subtypes. 8 machine learning methods revealed 8 hub genes with high accuracy and robustness (ALPL, ACTB, CD177, GAPDH, SLC25A37, S100A8, S100A9, and STXBP2). The SCENIC analysis revealed that APLP, CD177, GAPDH, S100A9, and STXBP2 were significant associated with various transcriptional factors. Finally, ALPL, CD177, S100A8, S100A9, and STXBP2 significantly up regulated in peripheral blood neutrophils of CLP and LPS-induced sepsis mice models.

**Conclusions:**

Our research discovered new clusters of neutrophils in sepsis. These five hub genes provide novel biomarkers targeting neutrophils for the treatment of sepsis.

## 1 Introduction

Sepsis is the result of a dysregulated host response to infection and can cause serious organ damage and even death. Mortality rates of sepsis rapidly exceed 30–35% without prompt and effective intervention (1). Over the past few decades, anti-inflammatory treatments have not been successful, and the immunosuppression often seen in patients with sepsis makes them more susceptible to nosocomial infections and organ dysfunctions (2). The causative factor for concomitant immunosuppression is lymphocyte exhaustion and reprogramming of innate immunity (3). Therefore, understanding the key mechanisms underlying sepsis-associated immune responses would lead to more potential therapeutic strategies (4).

Genome expression profiling studies have previously relied on whole blood to characterize diagnostic or prognostic genes, however, rarer cell types or cell type-specific signatures in sepsis were rarely detected (5). With the development of single-cell-RNA (sc-RNA) transcriptome, the immunosuppression condition was gradually discovered. It has been shown that myeloid cells are increased in number but lacking in function in sepsis (6). Guilin Li et al. reveals that the molecules CAP-1 and IL16 on monocytes may serve as potential diagnostic markers for sepsis based on sc-RNA analysis (7). Single-cell RNA sequencing (scRNA-seq) analysis revealed a new role of circulating MAIT17 in promoting sepsis severity and suggests the PI3K-LDHA signaling as a driving force in MAIT17 responses (8). By comparing the immune cell landscapes in sepsis with those in other conditions, researchers aim to identify common and disease-specific immune cell states and pathways, which could provide a more comprehensive understanding of the immune response and inform the development of novel therapeutic approaches.

Machine learning (ML) showed great promise in assessing high-dimensional data and identifying genes with biological significance (9). In sepsis, ML methods were widely used in identification of subclasses and development of prediction models (10, 11). Given sepsis is made up of multiple types of cells, such as neutrophils, macrophages, T cells and B cells, combination of single-cell sequencing and machine learning methods is proved to be a groundbreaking approach to study the genetic attributes of sepsis at the individual cell level (12).

In this study, we integrated single-cell RNA sequencing to identify specific neutrophils cell clusters and signature gene sets in sepsis. hdWGCNA (High Dimensional Weighted Correlation Network Analysis) method and multiple machine learning methods (Lasso, k-Nearest Neighbors, Linear Discriminant Analysis, Logistic Regression, Naive Bayes, Random Forest, Recursive Partitioning, and Support Vector Machine) were used to transcriptome analysis and hub genes identification. Finally, two mice models of sepsis were establishment and validated expressions of hub genes. Our findings provide an overall perspective on the heterogeneity of neutrophils in sepsis and will inform the development of personalized treatment and management strategies.

## 2 Methods

### 2.1 Patients and datasets

All datasets were obtained from the GEO dataset (https://www.ncbi.nlm.nih.gov/geo/). The scRNA-seq dataset GSE167363 included 2 healthy subsets and 4 sepsis subsets. The transcriptomic data were downloaded from GEO datasets GSE57065 and GSE95233. The training dataset GSE57065 included 25 healthy subsets and 28 sepsis subsets. The validation dataset GSE95233 included 22 healthy subsets and 51 sepsis subsets. GSE57065 and GSE95233 were also used for meta-analysis. Information of GSE167363, GSE57065, and GSE95233 were showed in **Supplementary Table S1**.

### 2.2 Single cell analysis

The R “Seurat” package (version 4.3.0.1) was used to process the scRNA-seq data. The expression profile was read in by Seurat package and screened out (nFeature_RNA>200 & nFeature_RNA<5000 & percent.mt < 20). 1Unsupervised clustering was conducted using Principal Component Analysis (PCA) and Uniform Manifold Approximation and Projection (UMAP) analysis after data normalization with the LogNormalize method, allowing us to see cell populations on a two-dimensional map. The Human Primary Cell Atlas was used as the source of reference material for the SingleR package’s cell annotation function. To identify each cluster’s marker gene, we used the “FindAllMarkers” tool and threshold values of fold change (FC).

### 2.3 High dimensional weighted correlation network analysis

High dimensional data, such as single cell RNA-seq, can be used to perform weighted gene co-expression network analysis (WGCNA) using hdWGCNA. hdWGCNA detects strong modules of linked genes and gives the biological context for these modules. It may be used to build co-expression networks in a cell type-specific manner. In this study, we investigated the hub genes of neutrophils under sepsis using hdWGCNA. And lastly, a subpopulation of hepatocytes undergoing regeneration was screened for distinctive genes. The “hdWGCNA” package version utilized in this research is 0.1.1.9010.

### 2.4 Trajectory analysis for cell subsets

The trajectory analysis was performed via the R “Monocle” package (version 2.18.0). The cell data set was constructed using an integrated gene expression matrix, exported from the Seurat object into Monocle. SetOrderingFilter was used to sequence cells based on the variable genes defined by dispersionTable. Finally, we then reduced the dimensionality of the trajectory using the Darter method and estimated the arrangement of cells using the orderCells function. The differentiation time of cell subsets was then identified clustering characteristics and gene markers.

### 2.5 Cellular communication analysis

R “CellChat” package (version 1.0.0) was used to analyze cellular communications. In sepsis, we investigated the differences and connections between immune cells during the current regeneration process, as well as the interactions of regeneration-related subsets with other cell types via cell communication analysis. Finally, we selected 4000 immune cells for processes.

### 2.6 Function analysis

High-throughput molecular research results are often translated into biological applications by analyzing gene function. We analyzed gene function using the R “clusterProfile” package (version 3.17) and visualized hub genes containing Disease Ontology (DO), Gene Ontology (GO) and Kyoto Encyclopedia of Genes and Genomes (KEGG). The significance level was set at *P*<0.05.

### 2.7 Identification of hub genes via machine learning algorithms

Multiple machine learning algorithms were applied to this study to identify feature genes. The first step was to perform Least Absolute Shrinkage and Selection Operator (Lasso) to screen candidates by iteratively reweighting least squares. Feature variables were selected based on minimum criteria after running the algorithm for 1000 cycles. Next, seven machine learning algorithms (k-Nearest Neighbors, Linear Discriminant Analysis, Logistic Regression, Naive Bayes, Random Forest, Recursive Partitioning, and Support Vector Machine) were applied. Finally, the receiver operating characteristic (ROC) curve was used to assess the classification performance of the hub genes in both training and validation cohorts. The pROC software was used to create ROC curves and the area under the curve was calculated.

### 2.8 Analysis of transcriptional factor regulatory network

Single-cell regulatory network interference and clustering (SCENIC, version 1.2.4) was created specifically for single cell data. Following the introduction of the gene co-expression network derived from transcription factor (TF) motifs by SCENIC, high-reliability gene regulatory networks (GRNs) predominately composed of TFs were found. We further highlighted the transcription factors of hub genes.

### 2.9 Immune infiltration analysis

CIBERSORT was used to determine immune cell proportions (13). Correlation analysis was then conducted to analyze the relationship between immune cells and eight hub genes (ACTB, ALPL, CD177, GAPDH, S100A8, S100A9, SLC25A37, and STXBP2).

### 2.10 GSEA analysis and ssGSEA analysis

Gene Set Enrichment Analysis (GSEA) (https://www.broadlnstitute.org/gsea/) and single sample Gene Set Enrichment Analysis (ssGSEA) were to explore the biological functions and gene expressions according to all gene sets in the Hallmark database.

### 2.11 Gene set variation analysis

The Gene Set Variation Analysis (GSVA) is an unsupervised, non-parametric method. Based on the R “GSVA” package (version 1.42.0) transformation of gene expression data, we analyzed the data as a feature derived from the expression matrix of each single gene. Rank statistics were used to calculate the gene sets for each feature, and expression matrices were converted into Enrichment Score (ES) matrixes. The GSVA enrichment score for each sample could be obtained, facilitating statistical analysis of the data.

### 2.12 Establishment of the sepsis mouse model

Cecal Ligation and Puncture (CLP) mice sepsis models: After inhaling isoflurane, eight-week C57 mice were anesthetized, and a stump was punctured with a 22-gauge needle once to expel stool. Afterward, the cecum was repositioned intraabdominally, and the abdomen closed. For fluid resuscitation, 0.2 ml of saline was administered intraperitoneally. Sham-operated mice did not undergo puncture or ligation. Mice were returned to cages and sacrificed 4 hours after the operation. The mice were intraperitoneally injected with phosphate-buffered saline (PBS) or Lipopolysaccharide (LPS) (20 mg/kg) to establish LPS-induced sepsis model.

### 2.13 Isolation of neutrophils

Mice of sepsis model were euthanized by cervical dislocation after injecting sodium pentobarbital intraperitoneally. Peripheral blood neutrophils were isolated from mice using the neutrophil isolation kit (130-097-658, Miltenyi).

### 2.14 Quantitative RealTime PCR

Following the manufacturer’s instructions, TRIzol reagent (12183-555, Invitrogen) was used to extract RNA from neutrophils. Reverse transcription was carried out using Takara’s Prime-Script Rase. After the premix Ex-Taq (Takara), the gene expression level was determined by qRT-PCR and normalized to the beta-actin (β-Actin). The expression level was calculated using the 2^-ΔΔCt^ technique. **Table 1** contains a list of the primer pairs utilized in the experiments. The study was approved by the Ethics Committee of General Hospital of Northern Theater.

**Table 1:** The primer sequence for PCR analysis.

Primer	Forward Sequence	Reverse Sequence
ACTB	ATTGTTACCAACTGGGACG	CTGGGTCATCTTTTCACG
ALPL	TCATCAGTATTTGGAAGAGC	GAGCGAAGGGTCAGTCAG
β-actin	GTGCTATGTTGCTCTAGACTTCG	ATGCCACAGGATTCCATACC
CD177	CCCCACCTATCAAACCTT	CAGATCCCAGCATACAAAG
GAPDH	AGGTCGGTGTGAACGGATTTG	GGGGTCGTTGATGGCAACA
S100A8	CCTCAGTTTGTGCAGAATAT	CCTTGTGGCTGTCTTTGT
S100A9	CGACACCTTCCATCAATAC	AACTGTGCTTCCACCATT
SLC25A37	AGACACGGATGCAGAGTT	TCATAGCAGGCAAAATACA
STXBP2	CCCACTATTACACGAACTCA	CTTCTTGGAAACATCTGCTA

### 2.15 Statistical analysis

To perform the statistical analysis, R software (version 4.2.3, available at https://www.r-project.org) and GraphPad Prism 8.0 software were used. The two-tailed Student t-test determined the statistical significance. Co-expression was adjusted using Pearson’s correlation. *P*<0.05 was deemed statistically significant.

## 3 Results

### 3.1 Flowchart of the study

**Figure 1:**
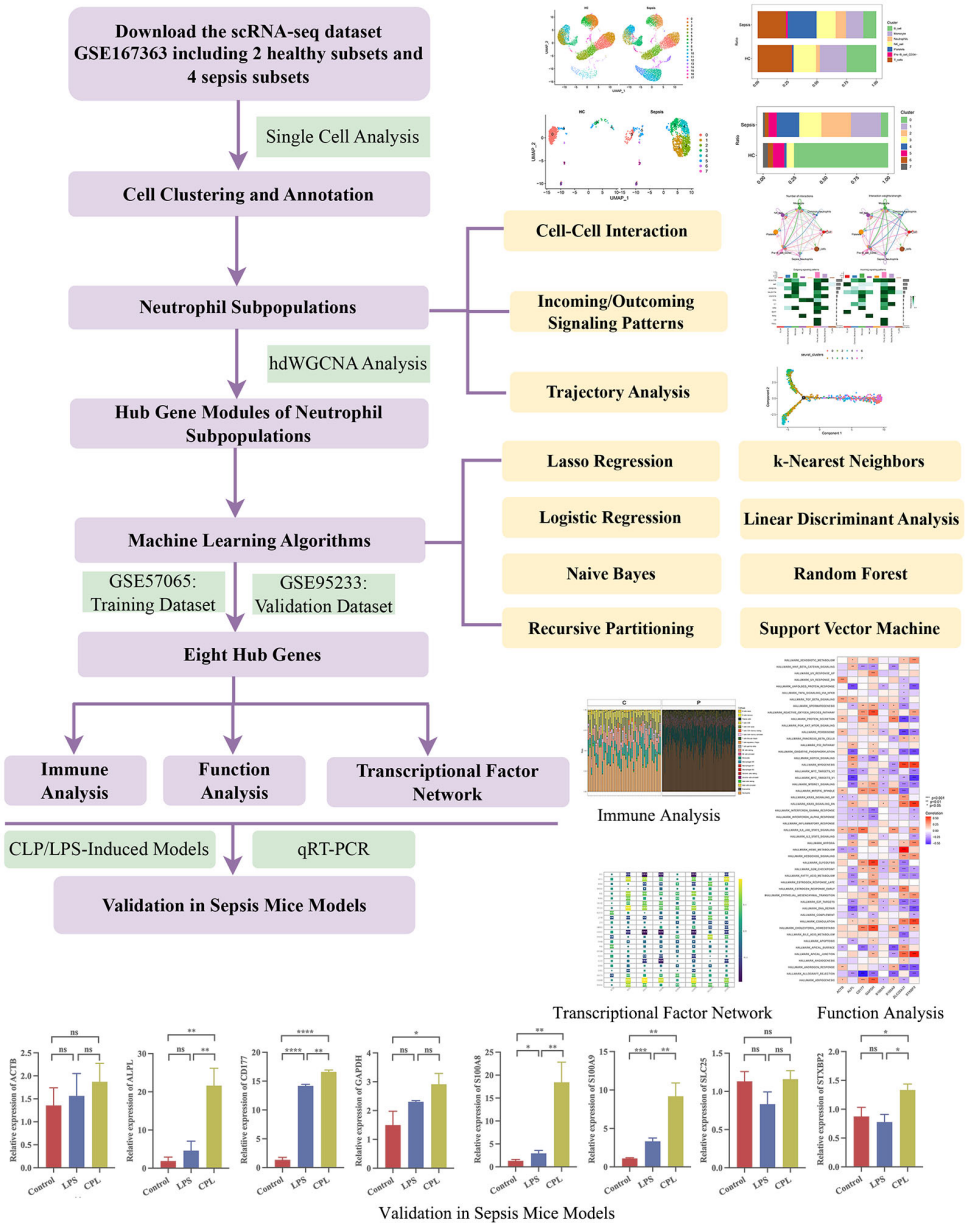
The flowchart of this study. *, P<0.05; **, P<0.01; ***, P<0.001; ****, P<0.0001; ns, P>0.05.

The flowchart of this study was showed in **Figure 1**.

### 3.2 Single-cell analysis reveals specific proportions of neutrophils

**Figure 2:**
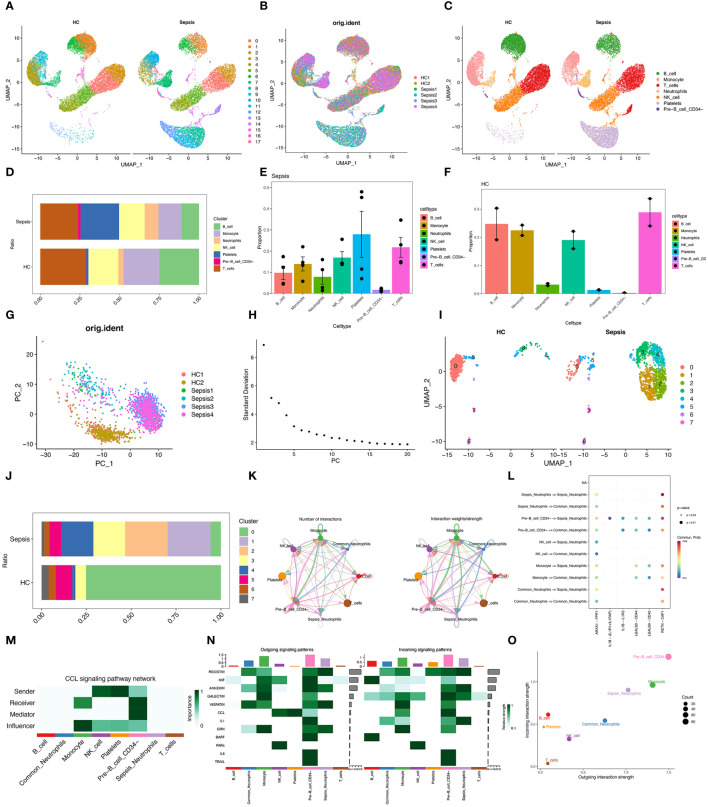
Single-cell analysis reveals specific proportions of neutrophils. **(A)** UMAP plots of immune cells in blood of healthy control and sepsis patients. **(B)** UMAP plots of two healthy control samples and four sepsis samples. **(C)** UMAP plots for single cell samples with different colors representing B cells, Monocyte, T cell, Neutrophils, NK cells, Platelets, and Pre-B CD34^-^ cells respectively. **(D)** Ration of B cells, Monocyte, T cell, Neutrophils, NK cells, Platelets, and Pre-B CD34^-^ cells in healthy control and sepsis patients. **(E, F)** Proportion of B cells, Monocyte, T cell, Neutrophils, NK cells, Platelets, and Pre-B CD34^-^ cells in healthy control and sepsis patients. **(G)** A PCA map of the distribution of cells in each sample, and each color represents the cells in each sample. **(H)** Elbowplot for identifying the optimal PCs. **(I)** UMAP plots of each neutrophil cluster in healthy control and sepsis patients. **(J)** Ration of each neutrophil cluster in healthy control and sepsis patients. **(K)** Number of interactions and interaction weights/strength between cell groups. **(L)** Bubble plots displayed the involved CCL-related signaling pathways documented in the “CellChat” R package in cell–cell communications. **(M)** The role of eight cell types in the CCL signaling pathway network. **(N)** Signaling role analysis on the aggregated cell–cell communication network from all signaling pathways between eight cell types. **(O)** The scatter plot of the inferred roles of eight cell type considering their ingoing and outgoing interaction strength.

To obtain high-quality cell samples from sepsis tissues, we preprocessed scRNA-seq data using stringent quality control metrics. We obtained 32,396 high-quality cell samples (**Supplementary Figure S1A**). The Pearson correlation coefficient of 0.82 showed a strong positive correlation between the number of genes detected and sequencing depth (**Supplementary Figure S1C**). The “Harmony” method and the “PCA” method was then used to reduce dimension and eliminate redundant samples (**Supplementary Figures S1B, D, E**). Unsupervised transcriptome analysis was performed using UMAP diagrams (**Figures 2A, B**). Cell abundance in healthy control and sepsis samples was showed (**Supplementary Figures S2A, B**). Seven separate clusters (B cells, Monocytes, T cells, Neutrophils, NK cells, Platelets, and CD34^-^ Pre-B cell) were showed on the UMAP diagram (**Figure 2C**). The ratio and proportion of those seven cell clusters in healthy control and sepsis samples were visualized (**Figures 2D–F**). In sepsis, Neutrophils, Platelets, and CD34-Pre-B cells tended to be increased, while other cell clusters (B cells, Monocytes, T cells, and NK cells) were on a decreasing trend. As neutrophils play a vital role in sepsis, our study then aimed to explore sepsis-related neutrophils. To explore the changes of neutrophils in sepsis, we first reduce dimension by the “PCA” method (**Figure 2G**). The ElbowPlot identified the optimal number of pcs as 11 (**Figure 2H**). UMAP diagram also showed abundances of neutrophils between sepsis and healthy controls (**Figure 2I**). Eight clusters of neutrophils were then identified. The ratio of eight separate clusters were also showed (**Figure 2J**). Cell numbers of each neutrophil cluster was showed in **Table 2**. Cluster 1&2 was not found in healthy controls, however, cluster 1&2 significantly increased in sepsis. Cluster 3&4 also increased in sepsis and cluster 0 significantly decreased in sepsis. The result indicated cluster 1&2 of neutrophils may be the potential responder cells against infection. It was found that Sepsis_Neutrophils-Monocyte and Sepsis_Neutrophils-Common_Neutrophils had higher interactions (**Figure 2K**). Moreover, interactions of Sepsis_Neutrophils-Monocyte and Sepsis_Neutrophils-Common_Neutrophils may rely on RETN-CAP1 signal transduction pathway (**Figure 2L**). However, Sepsis_Neutrophils showed no signifcant correlation with CCL signaling pathway, which plays an important role in immune response (**Figure 2M**). Furthermore, incoming, and outgoing signaling patterns analysis demonstrated that Sepsis_Neutrophils correlated with Resistin, Annexin, Visfatin, and IL1 (**Figure 2N**). Moreover, Sepsis_Neutrophils and Common_Neutrophils showed a significant difference on both incoming interaction strength and outgoing interaction strength (**Figure 2O**).

**Table 2:** Cell numbers of each neutrophil cluster in healthy control and sepsis samples.

Cluster	Cell numbers		
	Healthy control	Sepsis	Total
0	388	84	472
1	0	347	347
2	0	344	344
3	31	257	288
4	9	260	269
5	47	96	143
6	21	41	62
7	20	22	42

### 3.3 Pseudotime analysis and hdWGCNA analysis of sepsis-related neutrophils

**Figure 3:**
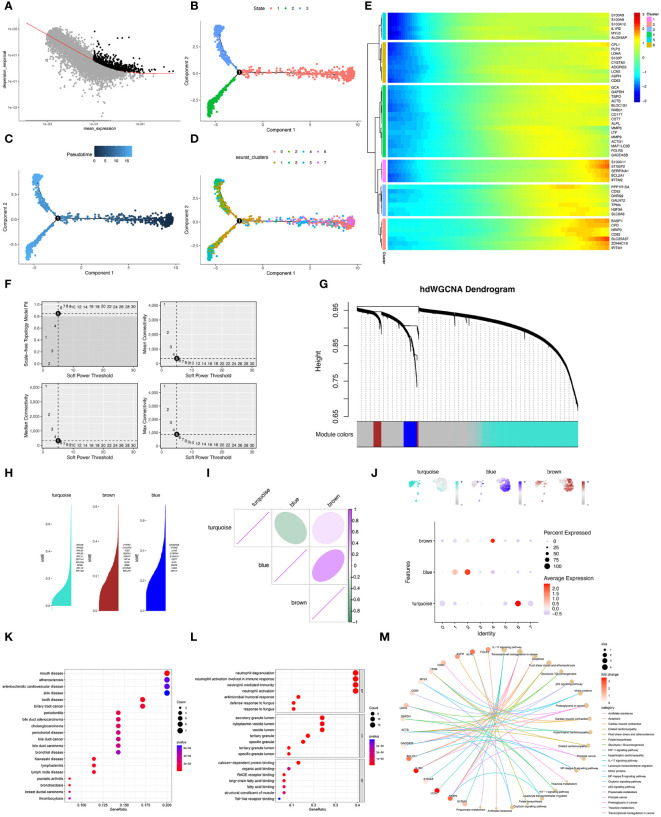
Pseudotime analysis and hdWGCNA analysis of sepsis-related neutrophils. **(A–D)** Pseudotime analysis for Neutrophils. **(E)** Six clusters of neutrophils by moncyte analysis and hub genes in each cluster were visualized. **(F)** Top left panel depicted the soft power threshold for choosing a scale-free topology model fit greater than or equal to 0.9. The other three panels showed the mean, median, and max connectivity of the topological network respectively when different minimum soft thresholds are chosen, reflecting the connectivity of the network. The average connectivity of the topological network is most stable at the lowest soft threshold equals. **(G)** Three modules were identified as shown in the hdWGCNA dendrogram. **(H)** Hub genes in each module were identified and ranked by k-means. **(I)** Correlation of three gene modules (turquoise, brown, and blue). **(J)** Feather plots depicted the corresponding module scores in neutrophils and the bubble plot displayed the scores obtained by three modules in neutrophils subtypes. **(K)** Dot plot of the DO functional enrich analysis of cluster 1&2 neutrophils. **(L)** Dot plot of the GO functional enrich analysis of cluster 1&2 neutrophils. **(M)** Cnet plot of the KEGG functional enrich analysis of cluster 1&2 neutrophils.

After standardization of data, the trajectory analysis projects all neutrophils cells onto three states (**Figures 3A, B**). The result showed that eight neutrophils’ clusters appeared in the pseudo-timeline, and the cluster 1&2 were most abundant in the state 2&3 (**Figures 3C, D**). The hub genes in cluster 1&2 were showed. Interestingly, all 50 hub genes all showed an elevated expression in the late stage of neutrophil-development (**Figure 3E**). This result was in consist of the trajectory analysis. Furthermore, hdWGCNA analysis was used for exploring gene modules related to cluster 1&2 of neutrophils. The soft threshold was adjusted to 5 for scale-free network construction (**Figure 3F**). Then the adjacency matrix and the TOM was built. Finally, 3 modules (Turquoise, brown, and blue modules) were identified based on average hierarchical clustering and dynamic tree clipping (**Figure 3G**). K-means methods identified hub genes in each module (**Figure 3H**). RPS18, RPS23, RPL32, RPL22, RPL11, EEF1A1, RPL23A, RPS8, RPL13, and RPL18A in the turquoise module. PTPRC, C16orf27, TLE3, ROCK1, H2AFY, HIF1A, KLF6, ZEB2, CTNNB1, and MALAT1 in brown module. GADD45B, IFITM2, LCN2, CYSTIM1, S100A12, CST7, ALPL, MMP9, CD63, and CD177 in blue module. The correlation analysis showed that the blue module was positively correlated with the brown module and negatively correlated with the turquoise module (**Figure 3I**). Moreover, among three modules, the blue module expressed highest in cluster 1&2 of neutrophils (**Figure 3J**). As cluster 1&2 of neutrophils may be the potential responder cells against infection, the genes in the blue module may have potential value for research. DO, GO, and KEGG enrichment analysis were performed on the genes in cluster 1&2 neutrophils (**Figures 3K–M**). The results showed that most of genes were enriched in the neutrophil degranulation, neutrophil activation, and neutrophil mediated immunity. IL-17 signaling pathway may be the potential mechanism in the neutrophil-related function. The pathway-network and pathway-gene interaction analyses were then showed.

### 3.4 Hub genes selection based on machine learning algorithms

**Figure 4:**
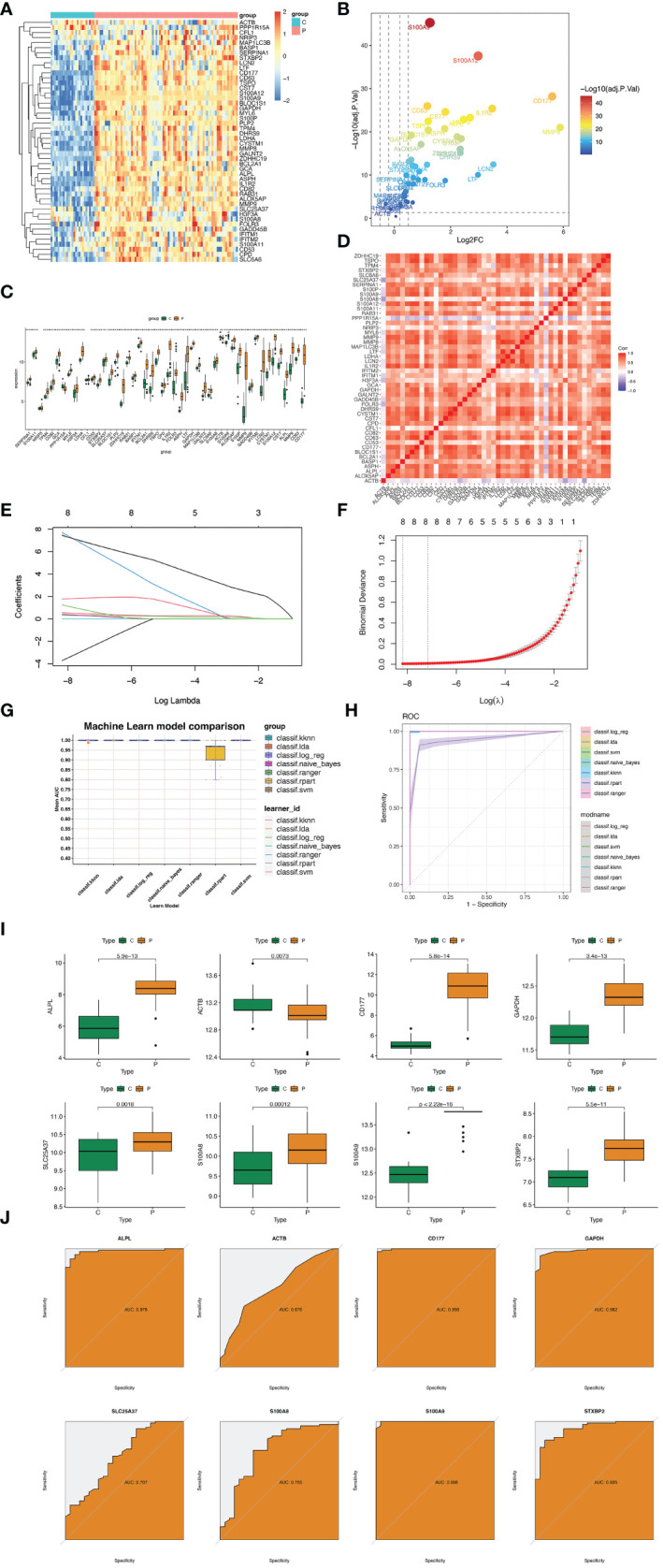
Hub genes selection based on machine learning algorithms. **(A)** Heatmap displaying the distribution of 50 DEGs. **(B)** Volcano plot showing expression of DEGs (|logFC|>1 and adjusted P value<0.05) in cluster 1&2 neutrophils. **(C)** Box plots displaying the expression of the expression of the top 50 genes. **(D)** Heatmap for correlations between 50 DEGs. **(E)** Coefficient profile plots showing the shrinkage of gene number. **(F)** Penalty plot for the LASSO model. **(G)** The AUC of seven machine learning althogrims. **(H)** Mean AUC of fivefold cross-validation for 9 replications of each model. **(I)** Expression difference of ALPL, ACTB, CD177, GAPDH, SLC25A37, S100A8, S100A9, and STXBP2 in GSE57056 between sepsis group and control group. **(J)** In GSE57056, ROC curve of predicted risk scores of ALPL, ACTB, CD177, GAPDH, SLC25A37, S100A8, S100A9, and STXBP2 in sepsis diagnosis. *, P<0.05.

GSE57065 was regarded as training dataset and GSE95233 was validation dataset. Firstly, these two datasets were normalized before analysis (**Supplementary Figures S3A–D**). Secondly, datasets GSE57065 and GSE95233 were merged and normalized (**Supplementary Figures S3E, F**). Thirdly, meta-analysis was used to eliminate the batch effect (**Supplementary Figures S3G, H**). The differences in expression of 50 hub genes were showed (**Figures 4A–C**). Moreover, correlations between every two genes were also identified (**Figure 4D**). Hub genes of cluster 1&2 Sepsis_Neutrophils were subjected to LASSO regression and 8 features were selected (ALPL, ACTB, CD177, GAPDH, SLC25A37, S100A8, S100A9, and STXBP2) (**Figures 4E, F**). The coefficient of each feature was listed as **Table 3**. Besides, seven machine learning algorithms (k-Nearest Neighbors, Linear Discriminant Analysis, Logistic Regression, Naive Bayes, Random Forest, Recursive Partitioning, and Support Vector Machine) were utilized and mean AUCs of seven algorithms were compared (**Figure 4G**). Only the Recursive Partitioning showed a lower AUC compared with other six algorithms (**Figure 4H**). Moreover, the AUC in the validation dataset was 0.988 and the model had a strong generalization ability (**Supplementary Figure S4**). We demonstrated the expression of eight hub genes in the training dataset GSE57065. All eight hub genes showed statical higher expressions in neutrophils under sepsis (**Figure 4I**). ROC curves showed that five hub genes (except ACTB, S100A8, and SLC5A37) all showed higher AUCs (**Figure 4J**). In validation dataset GSE95233, six hub genes except ACTB and SLC5A37 expressed higher significantly in neutrophils under sepsis (**Supplementary Figure S5A**). ROC curves showed that six hub genes except ACTB and SLC5A37 all showed higher AUCs (**Supplementary Figure S5B**).

**Table 3:** The coefficient of each feature.

Feature	Coefficient
ACTB	-3.7197339587957
ALPL	0.548896764272396
CD177	0.325706654849931
GAPDH	7.69112519608401
S100A8	0.400471605674625
S100A9	7.44130879698439
SLC25A37	1.77117069243124
STXBP2	1.24120863955331

### 3.5 Immune analysis of hub genes

**Figure 5:**
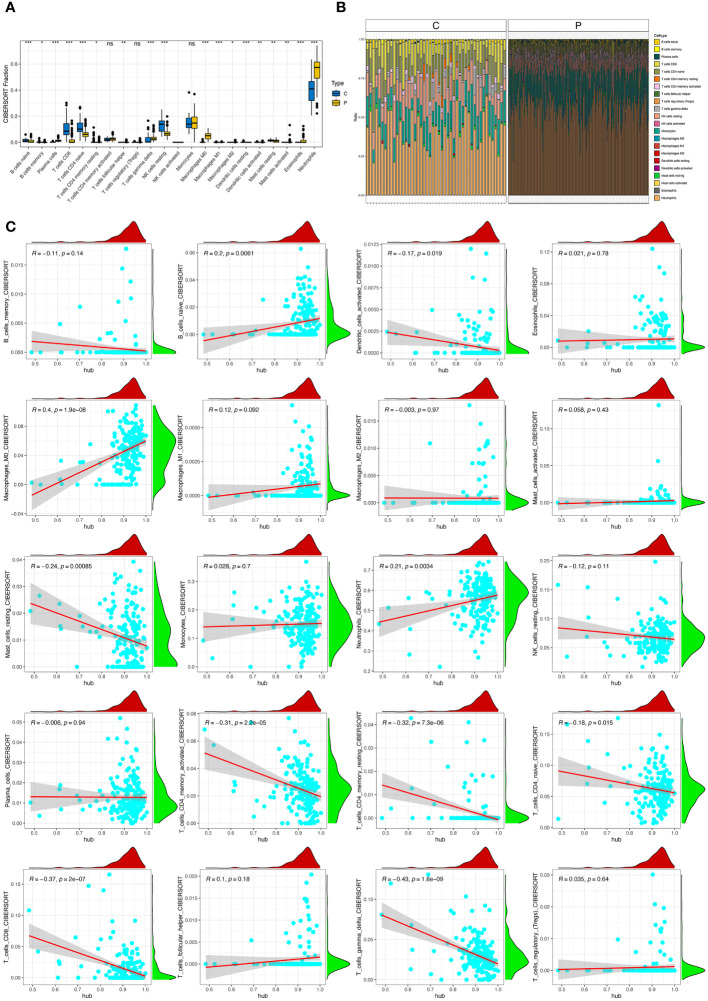
Immune infiltration in sepsis. **(A)** Panel representative boxplot shows the differences of infiltrated immune cells between sepsis samples (P type) and control samples (C type). **(B)** The relative proportions of 22 immune cell types between sepsis samples (P type) and control samples (C type). **(C)** Correlations between hub genes and immune cells. *, P<0.05; **, P<0.01; ***, P<0.001; ns, P>0.05.

Significant differences were observed in B cell naïve, B cells memory, Plasma cells, T cells CD8^+^, T cells CD4^+^ naïve, T cells CD4^+^ memory resting, T cells follicular helper, T cells gamma delta, NK cells resting, Macrophages M0, Macrophages M1, Macrophages M2, Dendritic cells resting, Dendritic cells activated, Mast cells resting, Mast cells activated, Eosinophils, and Neutrophils (**Figures 5A, B**). Moreover, the correlation between hub genes and immune cells were identified and visualized (**Figure 5C**). Hub genes were positively associated with Macrophage M0 cells. Hub genes negatively correlated with T cells CD4^+^ memory resting, T cells CD8^+^, T cells gamma delta, and T cells CD4^+^ memory activated. To explore the correlation between each hub gene and immune cells, correlation between 20 types of immune cells and eight hub genes were showed (**Supplementary Figures S6**–**S13**). Among eight hub genes, only S100A9 and STXBP2 showed no significant correlation with neutrophils. While correlations with other immune cells were also showed.

### 3.6 Function analysis of hub genes

**Figure 6:**
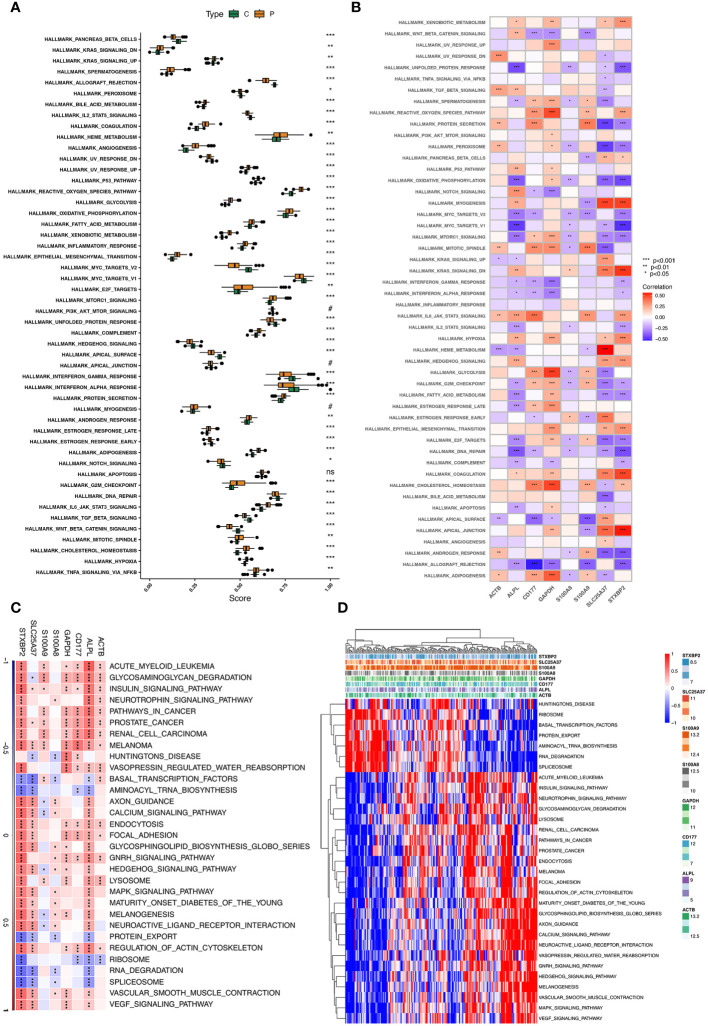
ssGSEA and GSVA of eight hub genes. **(A)** The specific distribution of the 50 hallmark gene sets in sepsis samples (P type) and control samples (C type). **(B)** Correlation analysis of the 50 hallmark gene sets with eight hub genes. **(C, D)** Heatmap showing the enriched pathways of eight hub genes in neutrophil clusters using GSVA.

ssGSEA and GSEA showed the potential biological pathway of eight hub genes in sepsis (**Figures 6A, B**). GSVA analysis showed that expression and correlation between eight hub genes and relative pathways in sepsis (**Figures 6C, D**). GSVA analysis showed that eight hub genes of neutrophils in sepsis were mainly enriched in Glycosaminoglycan_Degradation, Insulin_Signaling_Pathway, Prostate_Cancer, and Melanoma. Moreover, GSEA analysis reveals that related pathways of hub genes. Potential pathways that associated with up-regulation of eight hub genes(ALPL, ACTB, CD177, GAPDH, SLC25A37, S100A8, S100A9, and STXBP2)were showed in **Supplementary Figure S14A**. Results showed higher expression of eight hub genes mostly associated with starch and sucrose metabolism. Additionally, pathways of ribosome and graft-verus-host disease that associated with down-regulation of eight hub genes (ALPL, ACTB, CD177, GAPDH, SLC25A37, S100A8, S100A9, and STXBP2) were showed in **Supplementary Figure S14B**.

### 3.7 Transcriptional factor network and hub genes

**Figure 7:**
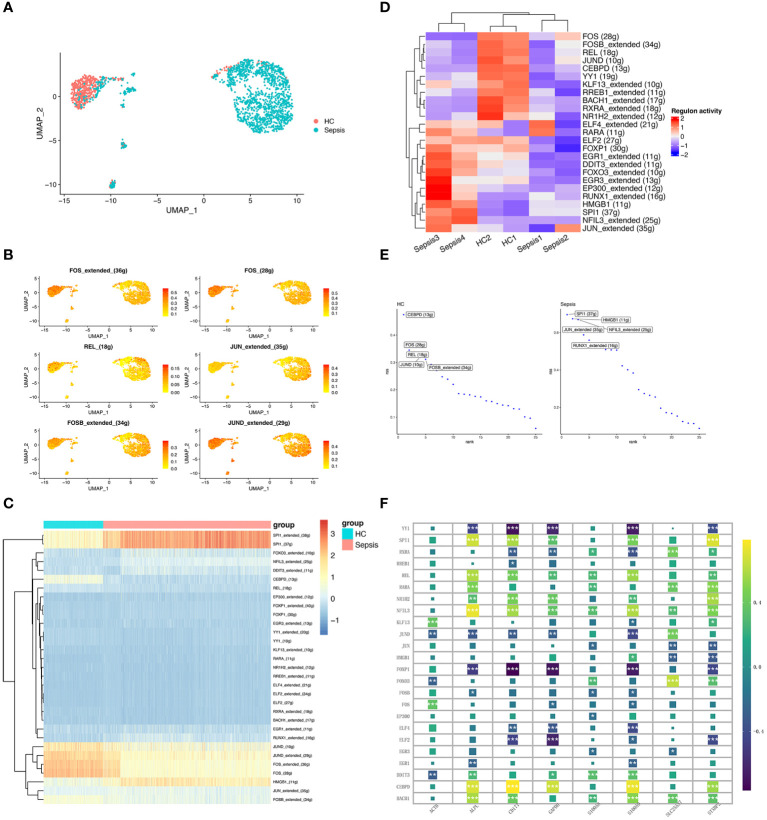
Transcriptional regulatory network of neutrophils. **(A)** UMAP plot depicts the distribution of neutrophils in the sepsis and healthy control groups. **(B)** Activity distribution of FOS, REL, JUN, FOSB, and JUND. **(C)** Heatmap shows the differences of TFs in neutrophils between the sepsis and healthy control groups. **(D)** Heatmap of the top 25 regulators with the highest area under curve (AUC) scores showing the activity of TFs in neutrophils clusters using SCENIC. **(E)** Top activities of TFs between different groups. RSS indicates Regulon Specificity Score. **(F)** Correlation between eight hub genes (ALPL, ACTB, CD177, GAPDH, SLC25A37, S100A8, S100A9, and STXBP2) expression and the level of 24 inferred TFs. *, P<0.05; **, P<0.01; ***, P<0.001.

SCENIC was to identify TFs with gene expression activity among subclusters of neutrophils. We applied the SCENIC analysis pipeline to the neutrophils and resolved distinct regulons associated with each cluster in neutrophils. Regulons were robust in activity and specific for each cell type (**Figures 7A, B**). For example, FOS_extended_(36g) and FOS_(28g)were lower active in the sepsis-specific neutrophils. Moreover, SPI1_extended_(38g), SPI1_(37g), and HMGB1_(11g) were highly active in the sepsis-specific neutrophils (**Figure 7C**). Heatmap of 25 TFs between sepsis and healthy control were showed (**Figure 7D**). The top ten TFs with the most difference between sepsis and healthy control, including SPI1, HMGB1, JUN, NFIL3, RUNX1, CEBPD, FOS, REL, JUND, and FOSB (**Figure 7E**). The correlation analysis of the inferred TFs and 8 hub genes was performed. APLP, CD177, GAPDH, S100A9, and STXBP2 were significant associated with various TFs (**Figure 7F**).

### 3.8 Validation of hub genes in sepsis mice model

**Figure 8:**
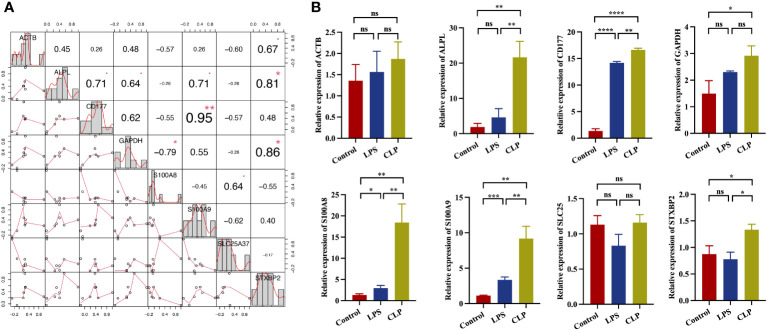
Correlations and the relative expressions of eight hub genes were validated by qRT-PCR. **(A)** Correlation between eight hub genes (ALPL, ACTB, CD177, GAPDH, SLC25A37, S100A8, S100A9, and STXBP2) expression. **(B)** The expressions of ALPL, ACTB, CD177, GAPDH, SLC25A37, S100A8, S100A9, and STXBP2 (ns, *P*>0.05; *P<0.05; **P<0.01; ***P<0.001; ****P<0.0001).

Moreover, gene correlations were identified and visualized (**Figure 8A**). CD177 and S100A9 showed a highest correlation with significance. To evaluate the expression of eight hub genes in neutrophils under sepsis. We established two sepsis model: CLP model and LPS-induced model. qRT-PCR detected the relative expression of all eight genes in monocytes. Results showed that ALPL, CD177, S100A8, S100A9, and STXBP2 significantly up regulated in sepsis (**Figure 8B**). In conclusion, ALPL, CD177, S100A8, S100A9, and STXBP2 may be play a vital role in neutrophils function response to sepsis.

## 4 Discussion

In this study, we first discovered new clusters of neutrophils between sepsis and healthy control samples based on the datasets GSE167363. A previous study showed the cells in GSE167363 were isolated within 24 h through density gradient centrifugation, which indicates the reliability of bioinformatics analysis (14). We identified 8 clusters of neutrophils based on sc-RNA analysis, while proportions of cluster 1&2 were with the most difference. Then, we applyied hdWGCNA, a thorough method for examining co-expression networks in highly dimensional transcriptomics data, to identify hub gene modules in new clusters 1&2 of neutrophils (15). Hub genes of new clusters of neutrophils were identified and the lasso regression screened eight hub genes (ACTB, ALPL, CD177, GAPDH, S100A8, S100A9, SLC25A37, and STXBP2). Seven machine learning algorithms (k-Nearest Neighbors, Linear Discriminant Analysis, Logistic Regression, Naive Bayes, Random Forest, Recursive Partitioning, and Support Vector Machine) were used to demonstrate the diagnostic performance. Then we investigated expression, the biological function, and the immune cell landscape of eight hub genes in sepsis. Finally, we validated the role of eight hub genes in both CLP- and LPS-induced sepsis in mice.

ML has shown great promise in enhancing the prediction, diagnosis, and management of sepsis. Min Huang et al. conducted the Support Vector Machine classifier and identified mortality biomarkers of sepsis (16). Moreover, another study applied a new machine learning method in predicting 20 differentially expressed genes for sepsis outcomes (17). In our study, we first applied single-cell RNA analysis to identify specific neutrophil clusters. Then, hdWGCNA and multiple machine learning methods were applied to identify hub genes. The hdWGCNA is an advanced computational framework designed to analyze and interpret high-dimensional transcriptomics data, such as single-cell RNA-seq or spatial transcriptomics (18). hdWGCNA was used in multiple cancer (including pancreatic cancer, breast cancer, and gastric cancer) (19–21). So far, hdWGCNA combining single-cell RNA sequencing has not been reported in sepsis. Moreover, we applied multiple ML algorithms to validate the clinical role of hub genes. This reflects the technique novelty of our study.

Neutrophils is an effector cell in the innate immune system that helps to fight infection as a first line of defense (22). Studies showed that sepsis-related neutrophils were dysfunctional and contributed to the multi-organ failure (22). Among multiple organs, the lung is the first and most frequently injured organ to fail (23). Moreover, the acute respiratory distress syndrome (ARDS), which resulted from the acute lung injury (ALI), was the main factors of sepsis patients’ deaths (23).

Neutrophils play both important protective and harmful functions in sepsis, according to data from animal models, some of which include particular subsets (24). Evidence has shown that different neutrophil subtypes or states, such as their release levels of cytokines, myeloperoxidase, reactive oxygen species (ROS), and neutrophil extracellular traps (NETs) in distinct clinical circumstances, are functionally diverse in critical immune phenotypes (25). Previous studies showed PD-L1 is upregulated on neutrophils during sepsis and neutrophils may suppress acquired immunity via the PD-L1/PD-1 immune checkpoint (26). The clinical observation of a “left shift” in the total blood count to more immature neutrophils in cases of severe illness in humans is well known (24). Hong et al. identify four neutrophil subtypes in sepsis based on sc-RNA sequencing and characterized by different expressing genes (27). In our study, we also identified sepsis-specific neutrophil clusters based on single-cell RNA analysis. Moreover, our work emphasized on revealing the genes that is specific to sepsis in those subpopulations of neutrophils based on “hdWGCNA” and multiple ML methods. However, our study showed that specific clusters of neutrophils appeared at the late stage of sepsis, which was in accord with the previous study (26). And these specific clusters may be the potential subsets of neutrophils in response to sepsis. Moreover, identifying specific clusters of neutrophils and related hub genes is vital for treating sepsis. RuiCi Lin. Et.al. found that Tram^-/-^ neutrophils enable effective reprogramming into a resolving state that is beneficial for treating experimental sepsis via reprogramming monocytes, neighboring neutrophils, T cells and endothelial cells (28). In our study, we identified two specific neutrophil subtypes and related eight hub genes (ACTB, ALPL, CD177, GAPDH, S100A8, S100A9, SLC25A37, and STXBP2). Indeed, only ALPL, CD177, S100A8, S100A9, and STXBP2 showed elevated expression *in vivo* sepsis model. Therefore, ALPL, CD177, S100A8, S100A9, and STXBP2 may be potential targets in treating sepsis.

Dysfunction of ALPL was known as the main cause of hypophosphatasia (HPP) (29). However, studies of ALPL in sepsis and neutrophils was still limited. In a prospective cross-sectional study containing 427 Emergency Department patients, ALPL showed as a biomarker of infectious and its AUC value was 0.83 (30). In neutrophils, ALPL encodes neutrophil alkaline phosphatase (NAP), a membrane-bound glycosylated protein that functions to catalyze dephosphorylation and transphosphorylation events (31). In sepsis, neutrophil NAP number substantially rises during bacterial infections when the cells are stimulated by inflammatory signals, and NAP-overexpressed neutrophils exhibit accelerated chemotaxis, which promotes their movement towards inflammatory areas, ROS production, and apoptosis (32).

CD177, a GPI-anchored protein, interacted with the proteinase-3 receptor (PR3) and CD177-PR3 complex regulates neutrophil migration in circulation (33). Previous study showed CD177 was up regulated in neutrophils under sepsis and neutrophil expressed CD177 anchored less with platelets, associated with less NETosis and worse outcome (34). Interestingly, expression of CD177 was negatively with expression of CD10 in neutrophils, which is a marker of immature myeloid cells (35). This was consisted with our study, which is those specific neutrophils with CD177 expression appeared at the late stage of sepsis. Ingred GR et al. identified a new cluster of neutrophils with high expression of CD177 and suppression of CD10 in response to infections (36). Moreover, this new cluster of neutrophils were found absence in healthy individuals and newly released from the bone marrow (36).

S100 family members S100A8 and S100A9 are cytoplasmic EF-hand Ca2^+^-binding proteins (37). A previous study verified that expressions of S100A8/A9 are elevated in blood cells of sepsis patients and S100A8/A9 showed high accuracy in sepsis diagnosis (38). S100A8/A9 also interacted with platelets under inflammation and regulated neutrophil recruitment (39). S100A8/A9 bind to TLR-4 and induce platelet pyroptosis, which are highly effective in causing NETosis (40). In addition, NETs release S100A8/A9, which further promotes platelet pyroptosis (40). They combine to generate a heterodimer that is strongly expressed in active neutrophils (37). Moreover, S100A8/A9 was mostly enriched in damage-associated molecular patterns (DAMPs) (41). Similarly, Sprenkeler EGG et al. showed that S100A8/A9 promote adhesion and elevate CD11b expression of neutrophils, which verify this DAMPs amplifies neutrophil activation (42). S100A9 also promotes neutrophils differentiated into pro-inflammatory N1 sub-population, as well as the chemotactic and enzymatic activity of N1 sub-population (43).

STXBP2, is crucial for the formation of the SNARE complex in platelets (44). In sepsis, STXBP2 in platelets regulated activation of platelets, NETosis, and sepsis thrombosis. However, the role of STXBP2 in neutrophils was still unclear. Our study showed that over-expressed of STXBP2 in neutrophils may be potential character in sepsis. Therefore, the specific function of STXBP2 needs to be further investigated.

## 5 Conclusions

In our study, we discovered specific clusters of neutrophils in sepsis based on single-cell RNA sequencing. We also identified five hub genes (ALPL, CD177, S100A8, S100A9, and STXBP2) in those special neutrophil clusters via the hdWGCNA method, machine learning algorithms, and transcriptomic analysis, as well as experimental verification. Our next step was to explore effects of five hub genes in neutrophils under sepsis. Five potential targets were identified for translational study in sepsis based on our novel mechanism.

## Data availability statement

The original contributions presented in the study are included in the article/**Supplementary Material**. Further inquiries can be directed to the corresponding author.

## Ethics statement

The animal studies were approved by Ethics Committee of General Hospital of Northern Theater Command. The studies were conducted in accordance with the local legislation and institutional requirements. Written informed consent was obtained from the owners for the participation of their animals in this study.

## Author contributions

PX: Writing – original draft. ZT: Writing – original draft. CZ: Writing – review & editing.

## Supplementary material

Supplementary Figure 1 Quality control of scRNA-seq data before analysis. **(A, B)** Quality control conditions. **(C)** Correlation between the number of genes detected and the sequencing depth. **(D)** The “PCA” method was used to reduce dimension. **(E)** The “harmony” method was used to eliminate redundant samples.

Supplementary Figure 2 Cell abundance of scRNA-seq samples. **(A, B)** Differential cell abundance from whole-blood scRNA-seq with sampled neighborhoods colored by statistical significance (spatial FDR < 0.05) in patient samples in 2 healthy subsets and 4 sepsis subsets.

Supplementary Figure 3 Data preprocessing. **(A)** Gene expression level statistics of GSE57056 before removed batch effect. **(B)** Gene expression level statistics of GSE57056 after removed batch effect. **(C)** Gene expression level statistics of GSE95233 before removed batch effect. **(D)** Gene expression level statistics of GSE95233 after removed batch effect. **(E)** Gene expression level statistics of meta datasets (merging GSE57056 and GSE95233) before removed batch effect. **(F)** Gene expression level statistics of meta datasets (merging GSE57056 and GSE95233) after removed batch effect. **(G-H)** PCA of merging data (GSE95233 and GSE57065).

Supplementary Figure 4 ROC curve of the diagnostic model in the independent external validation set.

Supplementary Figure 5 Identification of expression difference and ROC in the validation dataset GSE95233. **(A)** Expression difference of ALPL, ACTB, CD177, GAPDH, SLC25A37, S100A8, S100A9, and STXBP2 in GSE95233 between sepsis group and control group. **(B)** In GSE95233, receiver operating characteristic (ROC) curve of predicted risk scores of ALPL, ACTB, CD177, GAPDH, SLC25A37, S100A8, S100A9, and STXBP2 in sepsis diagnosis.

Supplementary Figure 6 The correlation between ALPL expression and immune model. **(A)** Correlation Coefficient between ALPL and 22 immune cell types. **(B)** Correlation between ALPL and immune cell types with significance.

Supplementary Figure 7 The correlation between ACTB expression and immune model. **(A)** Correlation Coefficient between ACTB and 22 immune cell types. **(B)** Correlation between ACTB and immune cell types with significance.

Supplementary Figure 8 The correlation between CD177 expression and immune model. **(A)** Correlation Coefficient between CD177 and 22 immune cell types. **(B)** Correlation between CD177 and immune cell types with significance.

Supplementary Figure 9 The correlation between GAPDH expression and immune model. **(A)** Correlation Coefficient between GAPDH and 22 immune cell types. **(B)** Correlation between GAPDH and immune cell types with significance.

Supplementary Figure 10 The correlation between S100A8 expression and immune model. **(A)** Correlation Coefficient between S100A8 and 22 immune cell types. **(B)** Correlation between S100A8 and immune cell types with significance.

Supplementary Figure 11 The correlation between S100A9 expression and immune model. **(A)** Correlation Coefficient between S100A9 and 22 immune cell types. **(B)** Correlation between S100A9 and immune cell types with significance.

Supplementary Figure 12 The correlation between SLC25A17 expression and immune model. **(A)** Correlation Coefficient between SLC25A17 and 22 immune cell types. **(B)** Correlation between SLC25A17 and immune cell types with significance.

Supplementary Figure 13 The correlation between STXBP2 expression and immune model. **(A)** Correlation Coefficient between STXBP2 and 22 immune cell types. **(B)** Correlation between STXBP2 and immune cell types with significance.

Supplementary Figure 14 GSEA identifies signaling pathways in the optimal hub genes. **(A)** The main signaling pathways that are significantly enriched under the up regulation of ALPL, ACTB, CD177, GAPDH, SLC25A37, S100A8, S100A9, and STXBP2. **(B)** The main signaling pathways that are significantly enriched under the downregulation of ALPL, ACTB, CD177, GAPDH, SLC25A37, S100A8, S100A9, and STXBP2.

## Glossary

**Table:**

sc-RNA	Single-cell-RNA
ML	Machine Learning
hdWGCNA	High Dimensional Weighted Correlation Network Analysis
PCA	Principal Component Analysis
UMAP	Uniform Manifold Approximation and Projection
WGCNA	Weighted Gene Co-expression Network Analysis
DO	Disease Ontology
GO	Gene Ontology
KEGG	Kyoto Encyclopedia of Genes and Genomes
ROC	Receiver Operating Characteristic
SCENIC	Single-cell Regulatory Network Interference and Clustering
TF	Transcription Factor
GSEA	Gene Set Enrichment Analysis
ssGSEA	single sample Gene Set Enrichment Analysis
GSVA	Gene Set Variation Analysis
ES	Enrichment Score
CLP	Cecal Ligation and Puncture
PBS	Phosphate-Buffered Saline
LPS	Lipopolysaccharide
qRT-PCR	Quantitative Real Time-PCR
β-Actin	Beta-Actin
ARDS	Acute Respiratory Distress Syndrome
ALI	Acute Lung Injury
ROS	Reactive Oxygen Species
NETs	Neutrophil Extracellular Traps
HPP	Hypophosphatasia
NAP	Neutrophil Alkaline Phosphatase
DAMPs	Damage-associated Molecular Patterns
AUC	Area Under Curve
