# Supplementary figures and images for: Integrated multi-omics and artificial intelligence to explore new neutrophils clusters and potential biomarkers in sepsis with experimental validation
Source: Front Immunol. 2024 May 29;15:1377817. doi: 10.3389/fimmu.2024.1377817 (PMC11167131; doi:10.3389/fimmu.2024.1377817)

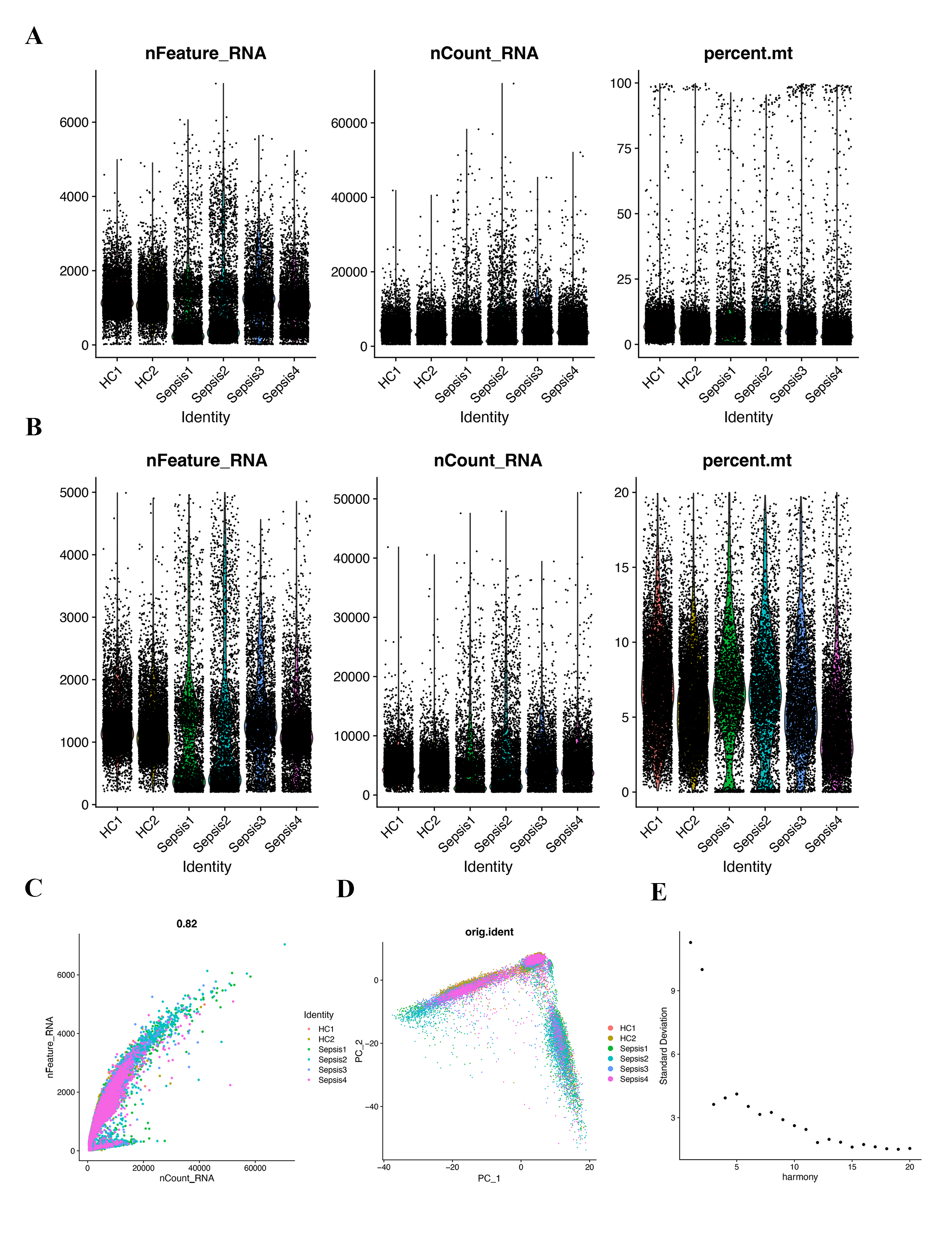

Supplement: Supplementary Figure 1 — Quality control of scRNA-seq data before analysis. (A, B) Quality control conditions. (C) Correlation between the number of genes detected and the sequencing depth. (D) The “PCA” method was used to reduce dimension. (E) The “harmony” method was used to eliminate redundant samples. [file Image_1.tif]

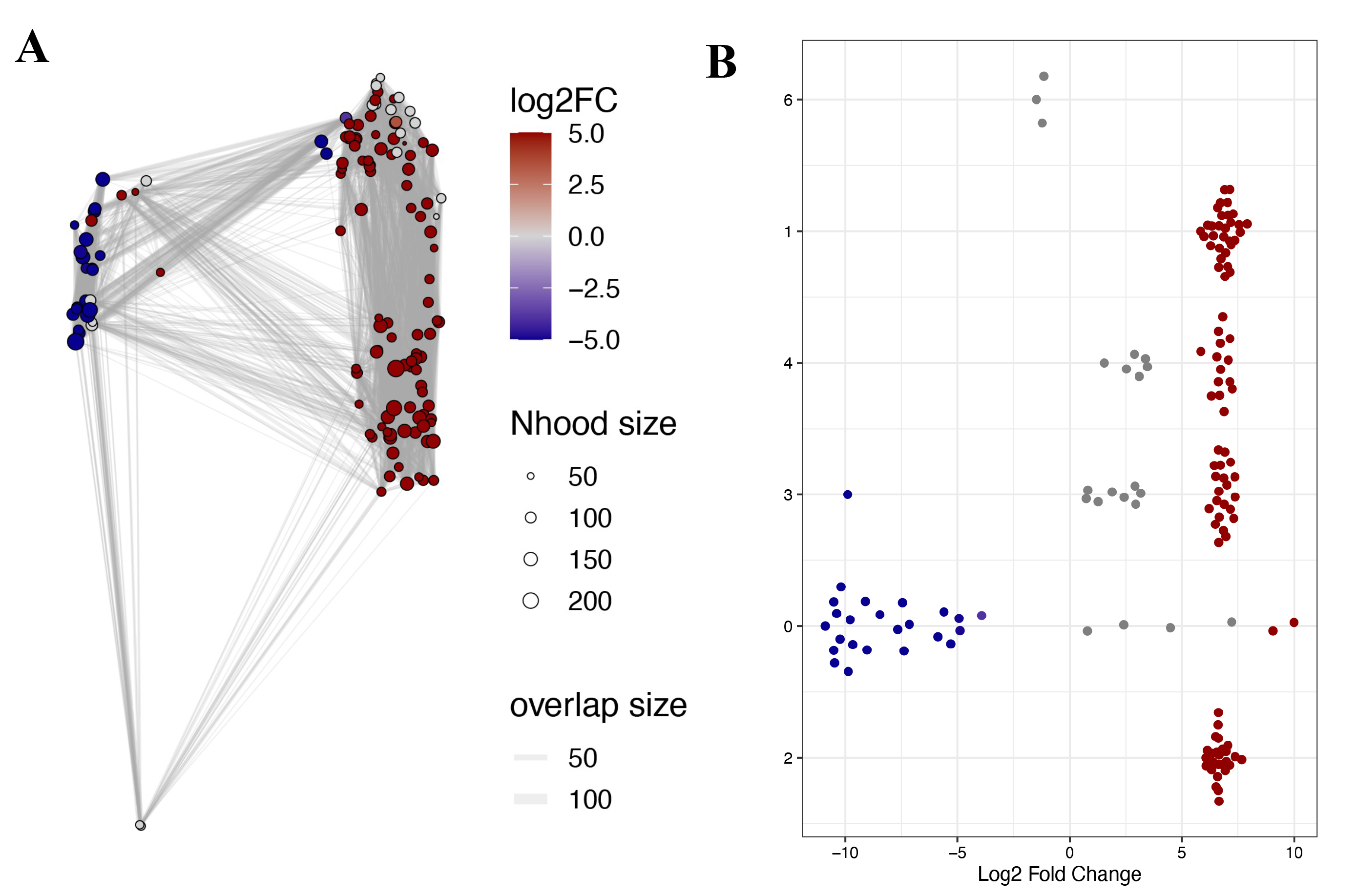

Supplement: Supplementary Figure 2 — Cell abundance of scRNA-seq samples. (A, B) Differential cell abundance from whole-blood scRNA-seq with sampled neighborhoods colored by statistical significance (spatial FDR < 0.05) in patient samples in 2 healthy subsets and 4 sepsis subsets. [file Image_2.tif]

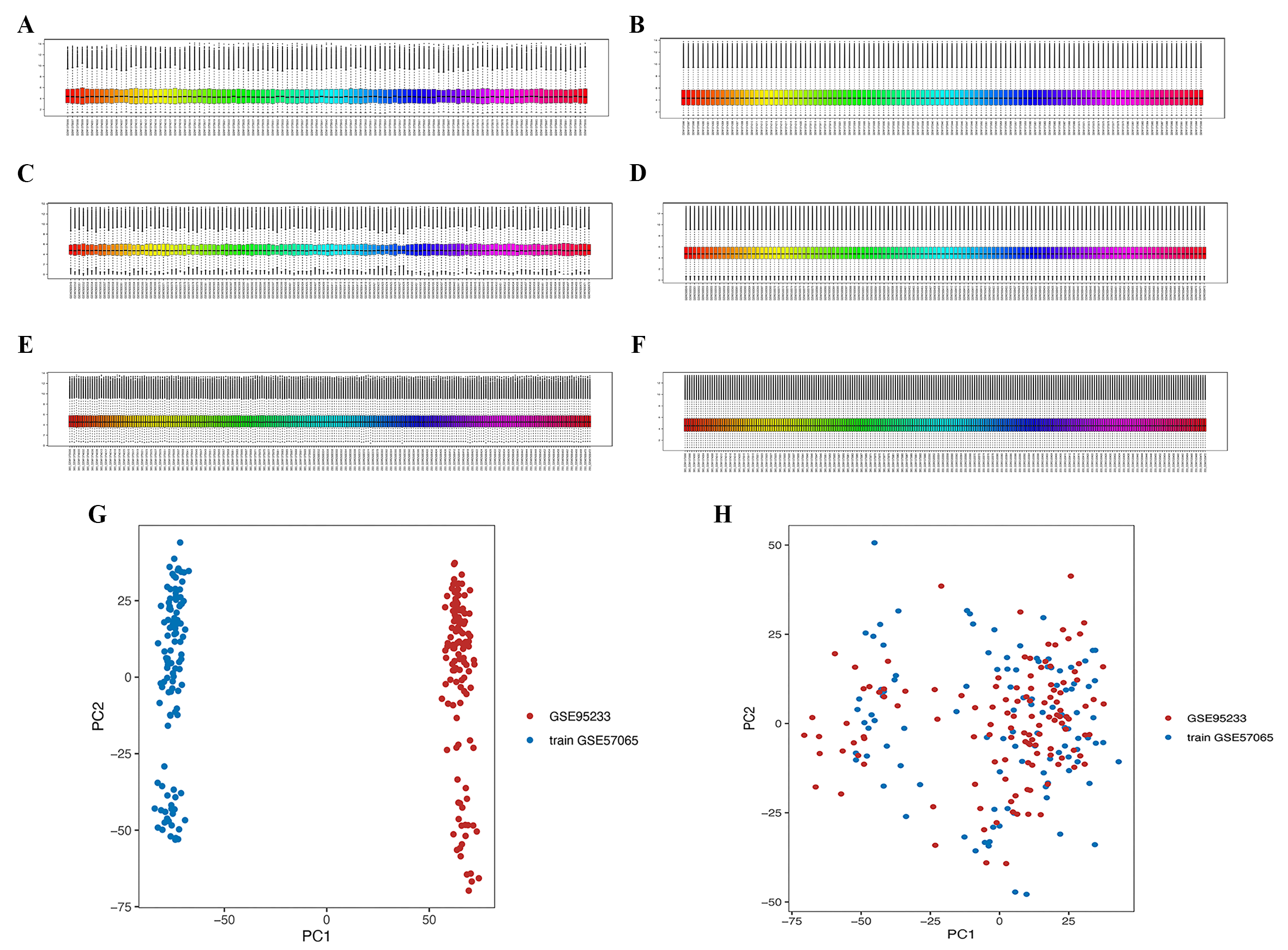

Supplement: Supplementary Figure 3 — Data preprocessing. (A) Gene expression level statistics of GSE57056 before removed batch effect. (B) Gene expression level statistics of GSE57056 after removed batch effect. (C) Gene expression level statistics of GSE95233 before removed batch effect. (D) Gene expression level statistics of GSE95233 after removed batch effect. (E) Gene expression level statistics of meta datasets (merging GSE57056 and GSE95233) before removed batch effect. (F) Gene expression level statistics of meta datasets (merging GSE57056 and GSE95233) after removed batch effect. (G-H) PCA of merging data (GSE95233 and GSE57065). [file Image_3.tif]

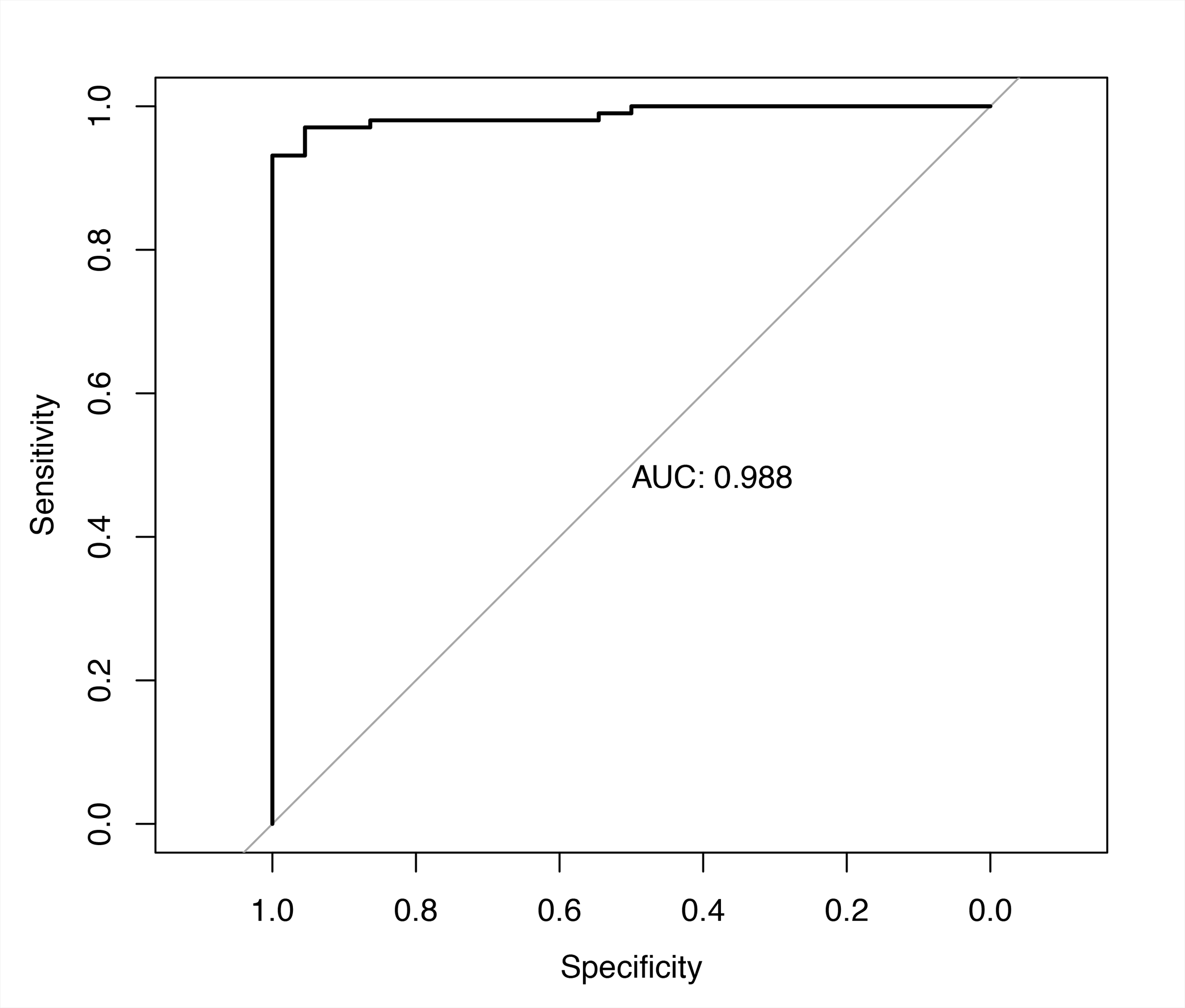

Supplement: Supplementary Figure 4 — ROC curve of the diagnostic model in the independent external validation set. [file Image_4.tif]

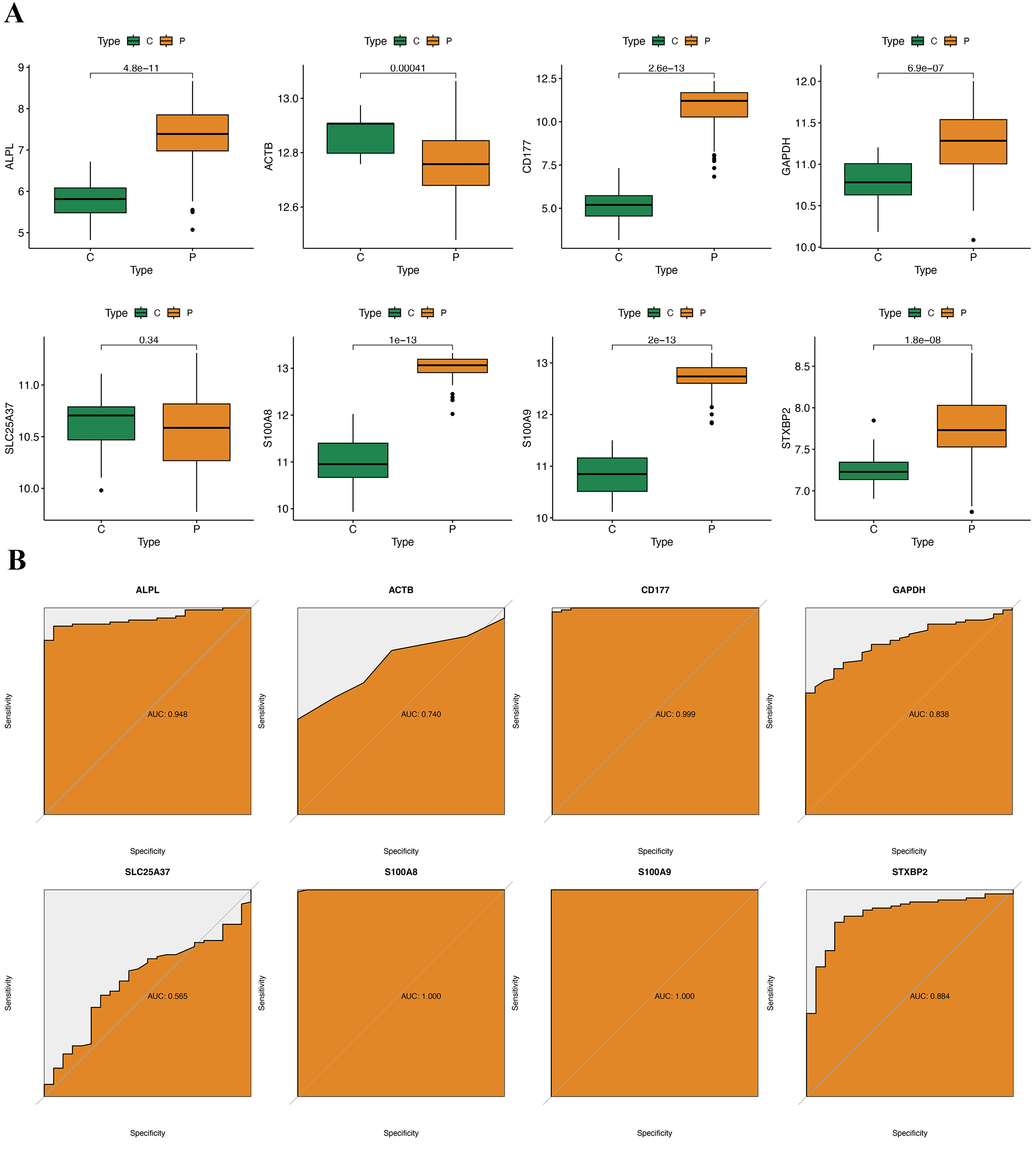

Supplement: Supplementary Figure 5 — Identification of expression difference and ROC in the validation dataset GSE95233. (A) Expression difference of ALPL, ACTB, CD177, GAPDH, SLC25A37, S100A8, S100A9, and STXBP2 in GSE95233 between sepsis group and control group. (B) In GSE95233, receiver operating characteristic (ROC) curve of predicted risk scores of ALPL, ACTB, CD177, GAPDH, SLC25A37, S100A8, S100A9, and STXBP2 in sepsis diagnosis. [file Image_5.tif]

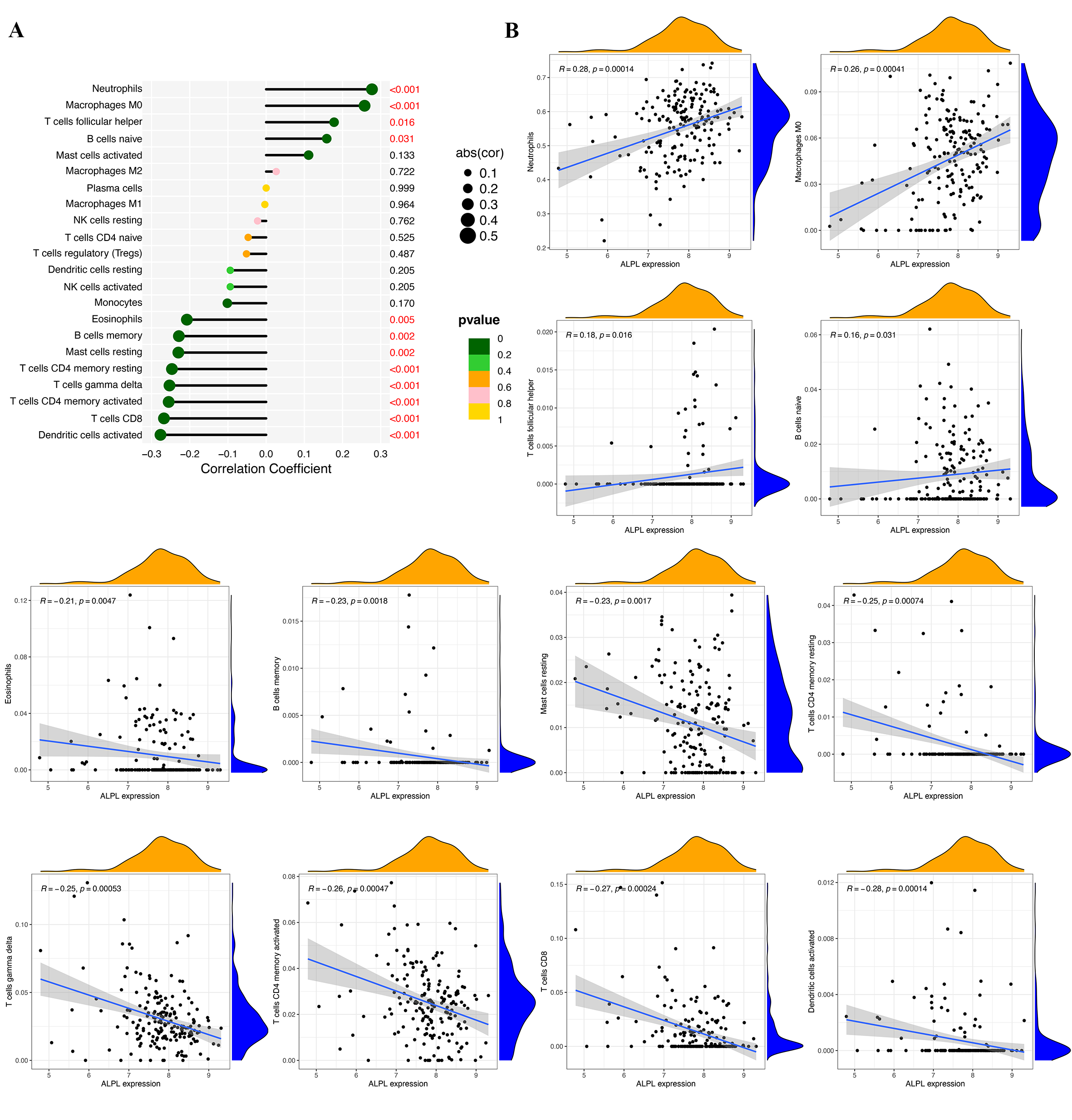

Supplement: Supplementary Figure 6 — The correlation between ALPL expression and immune model. (A) Correlation Coefficient between ALPL and 22 immune cell types. (B) Correlation between ALPL and immune cell types with significance. [file Image_6.tif]

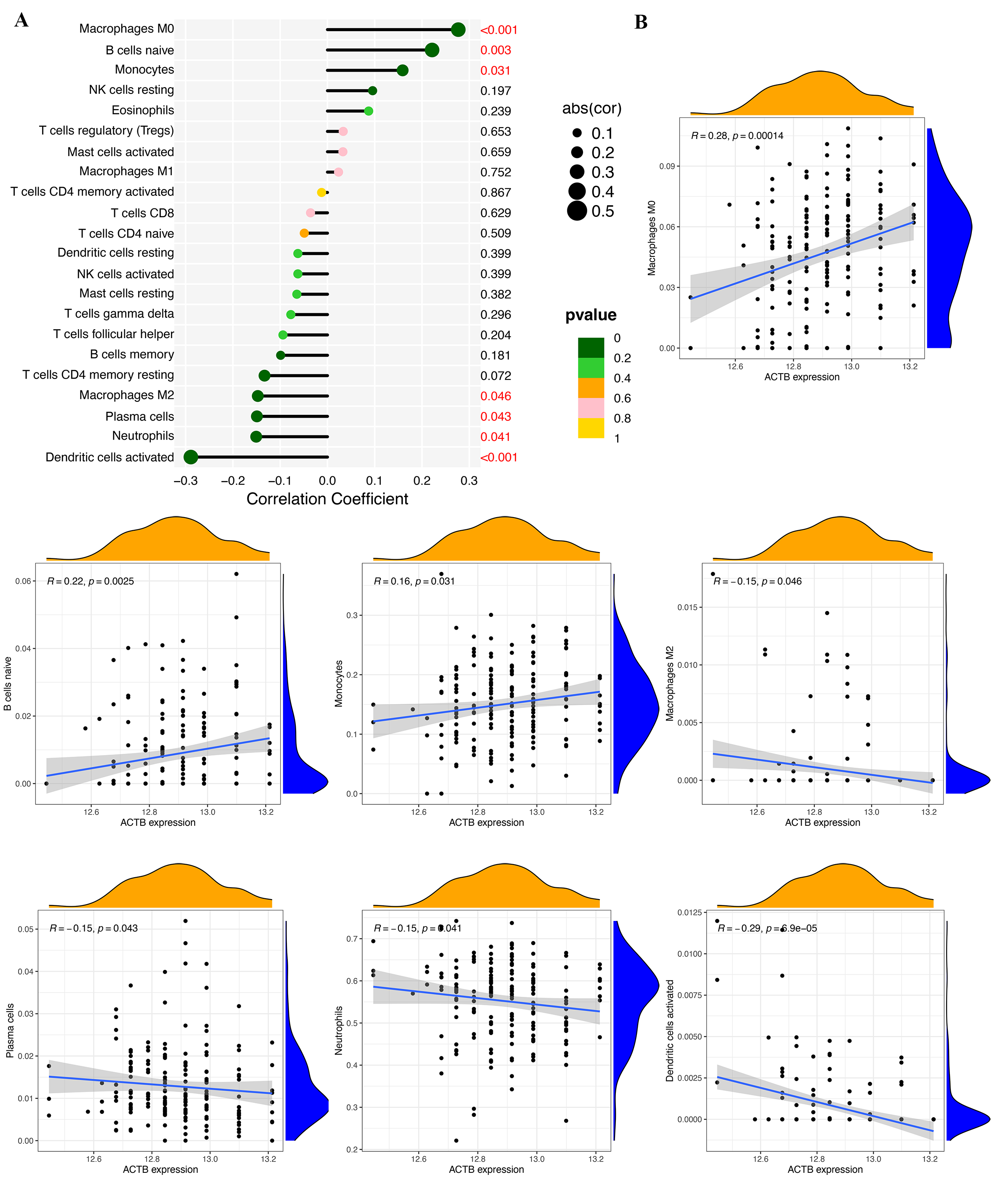

Supplement: Supplementary Figure 7 — The correlation between ACTB expression and immune model. (A) Correlation Coefficient between ACTB and 22 immune cell types. (B) Correlation between ACTB and immune cell types with significance. [file Image_7.tif]

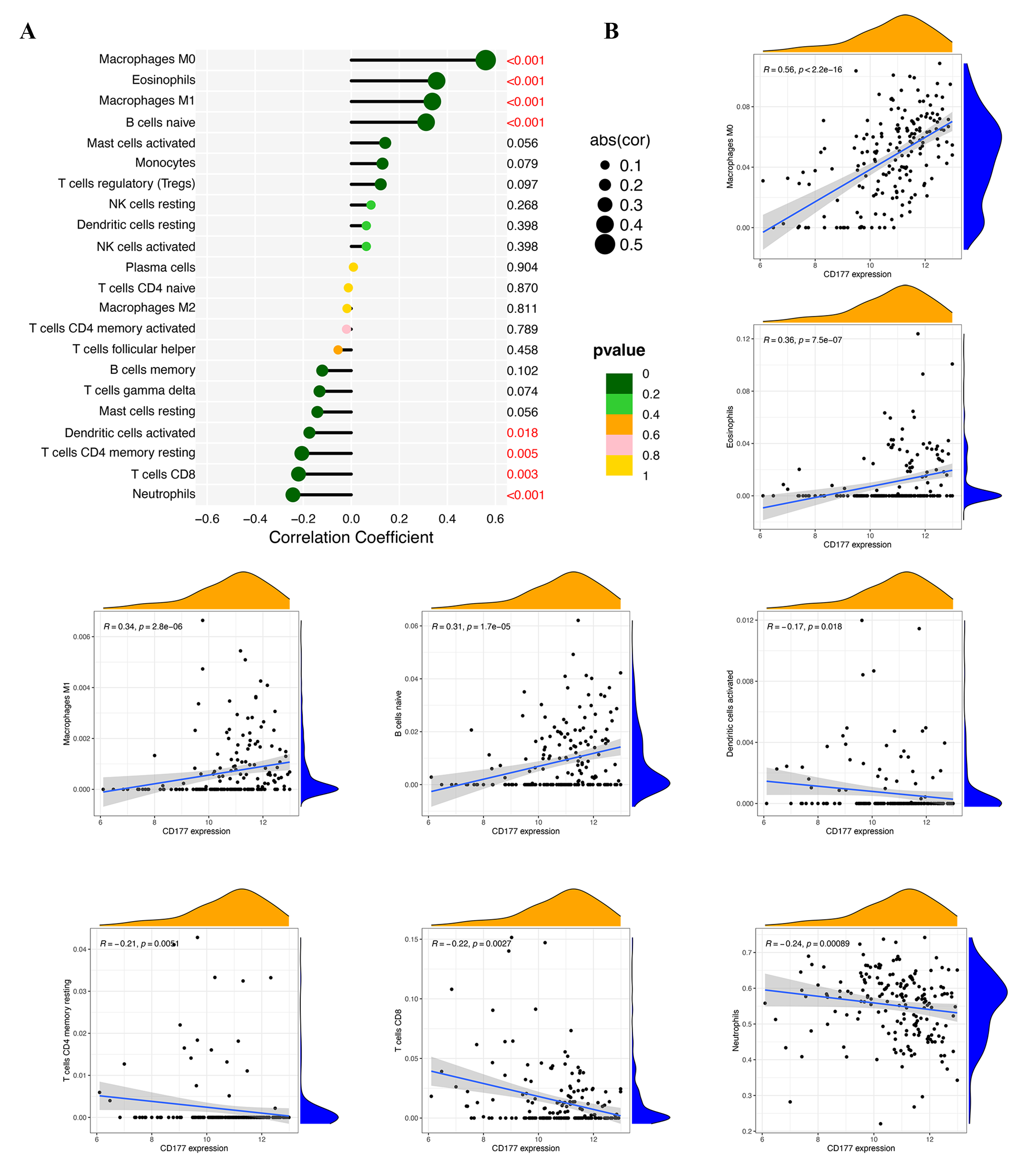

Supplement: Supplementary Figure 8 — The correlation between CD177 expression and immune model. (A) Correlation Coefficient between CD177 and 22 immune cell types. (B) Correlation between CD177 and immune cell types with significance. [file Image_8.tif]

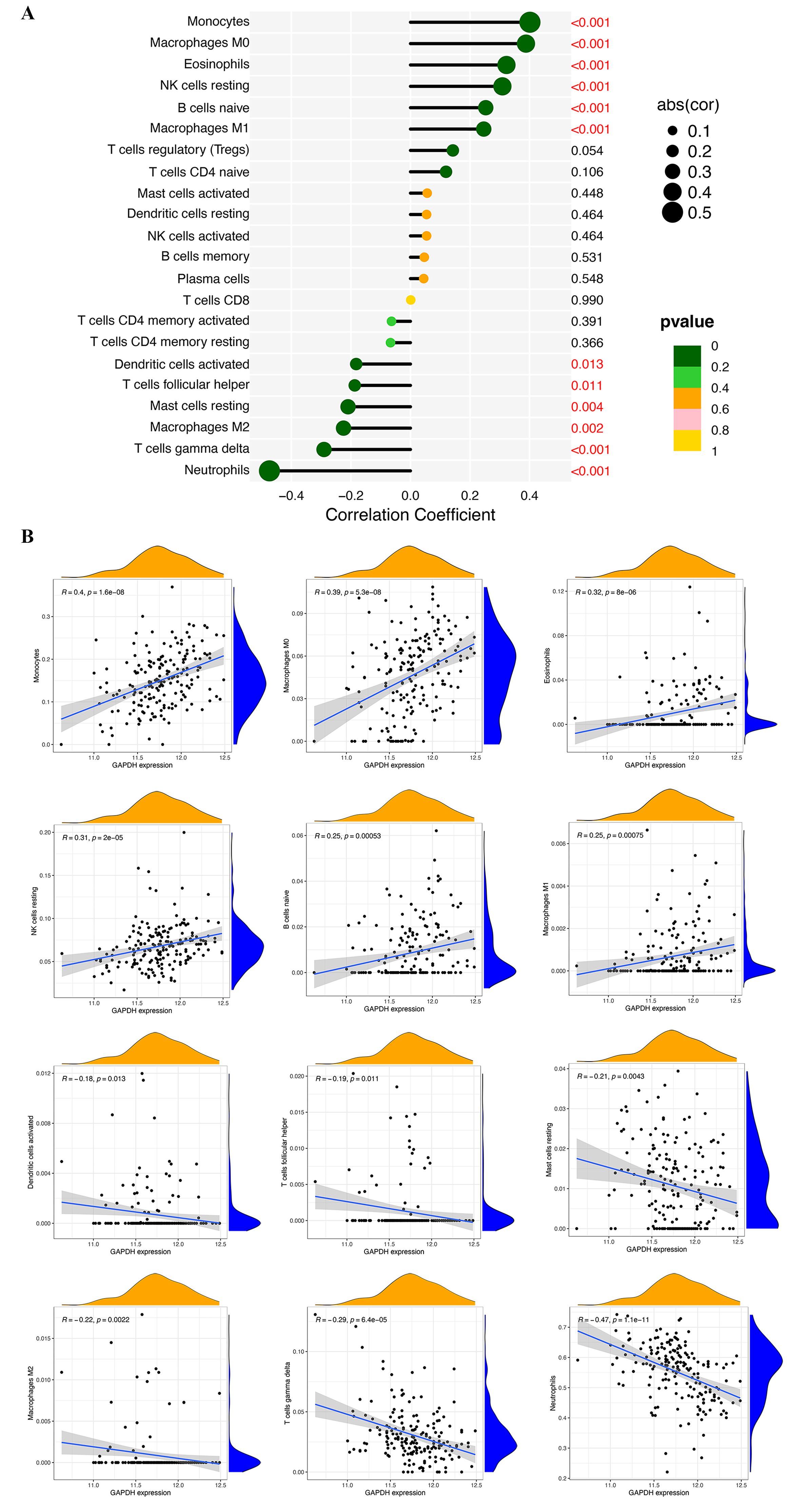

Supplement: Supplementary Figure 9 — The correlation between GAPDH expression and immune model. (A) Correlation Coefficient between GAPDH and 22 immune cell types. (B) Correlation between GAPDH and immune cell types with significance. [file Image_9.tif]

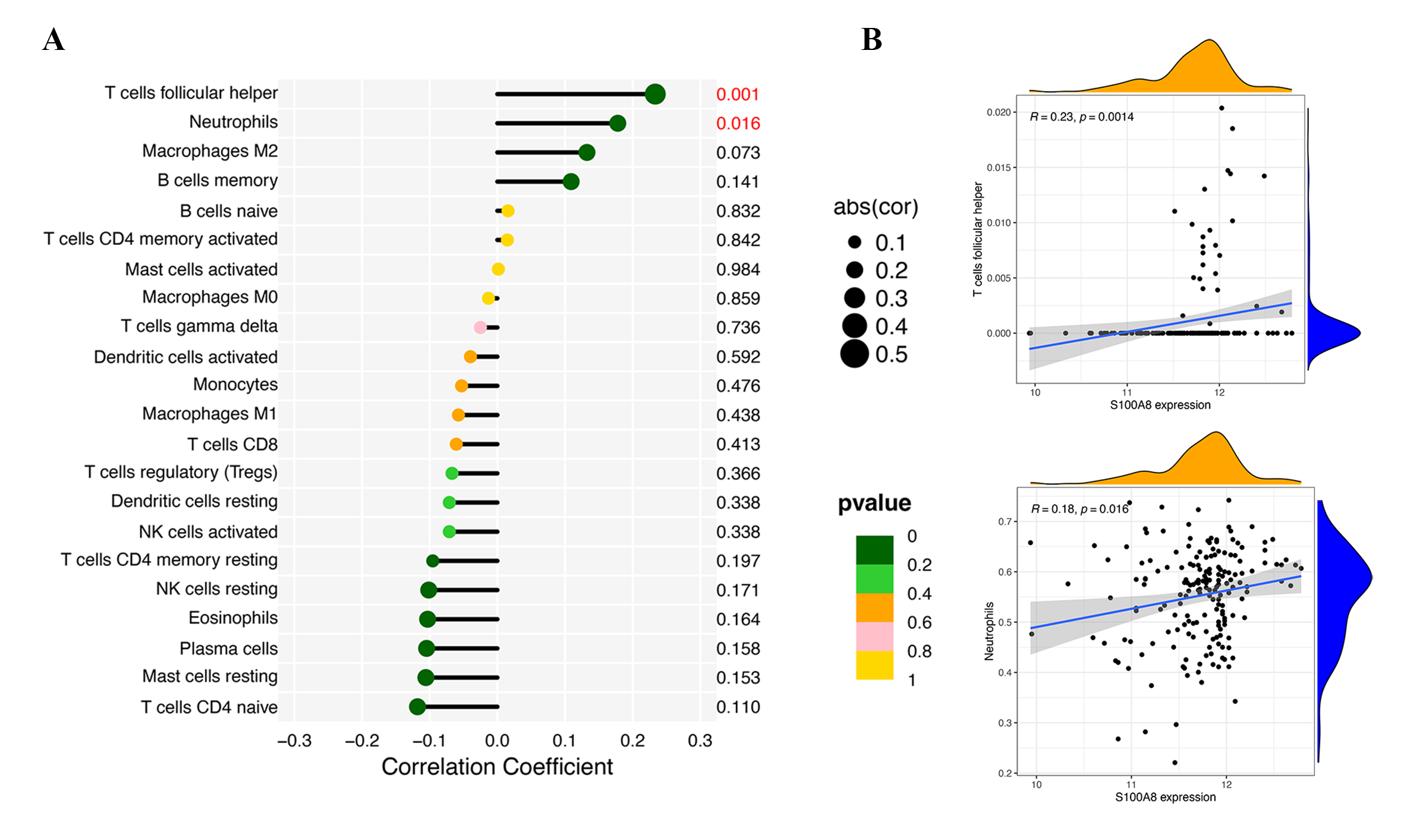

Supplement: Supplementary Figure 10 — The correlation between S100A8 expression and immune model. (A) Correlation Coefficient between S100A8 and 22 immune cell types. (B) Correlation between S100A8 and immune cell types with significance. [file Image_10.tif]

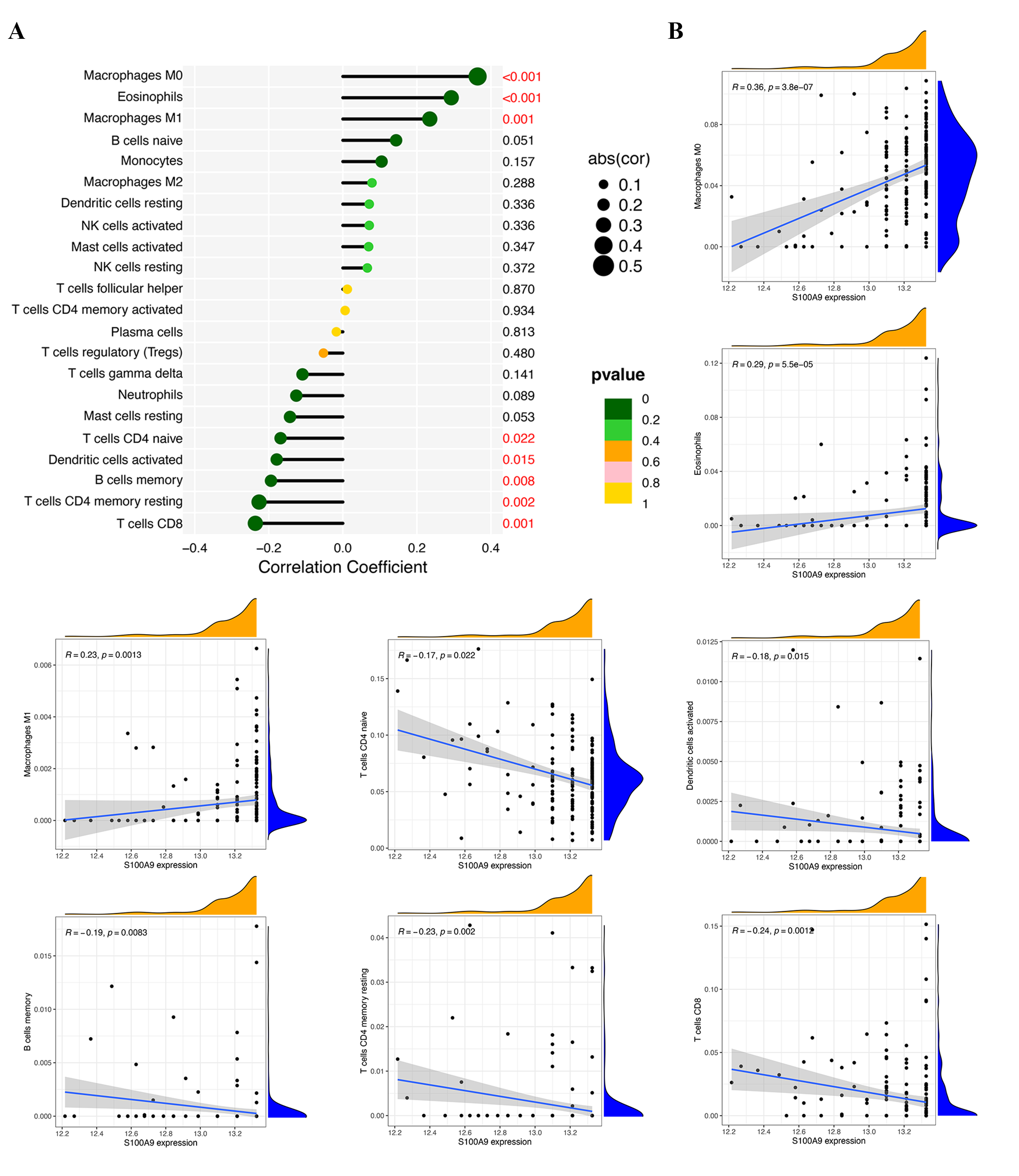

Supplement: Supplementary Figure 11 — The correlation between S100A9 expression and immune model. (A) Correlation Coefficient between S100A9 and 22 immune cell types. (B) Correlation between S100A9 and immune cell types with significance. [file Image_11.tif]

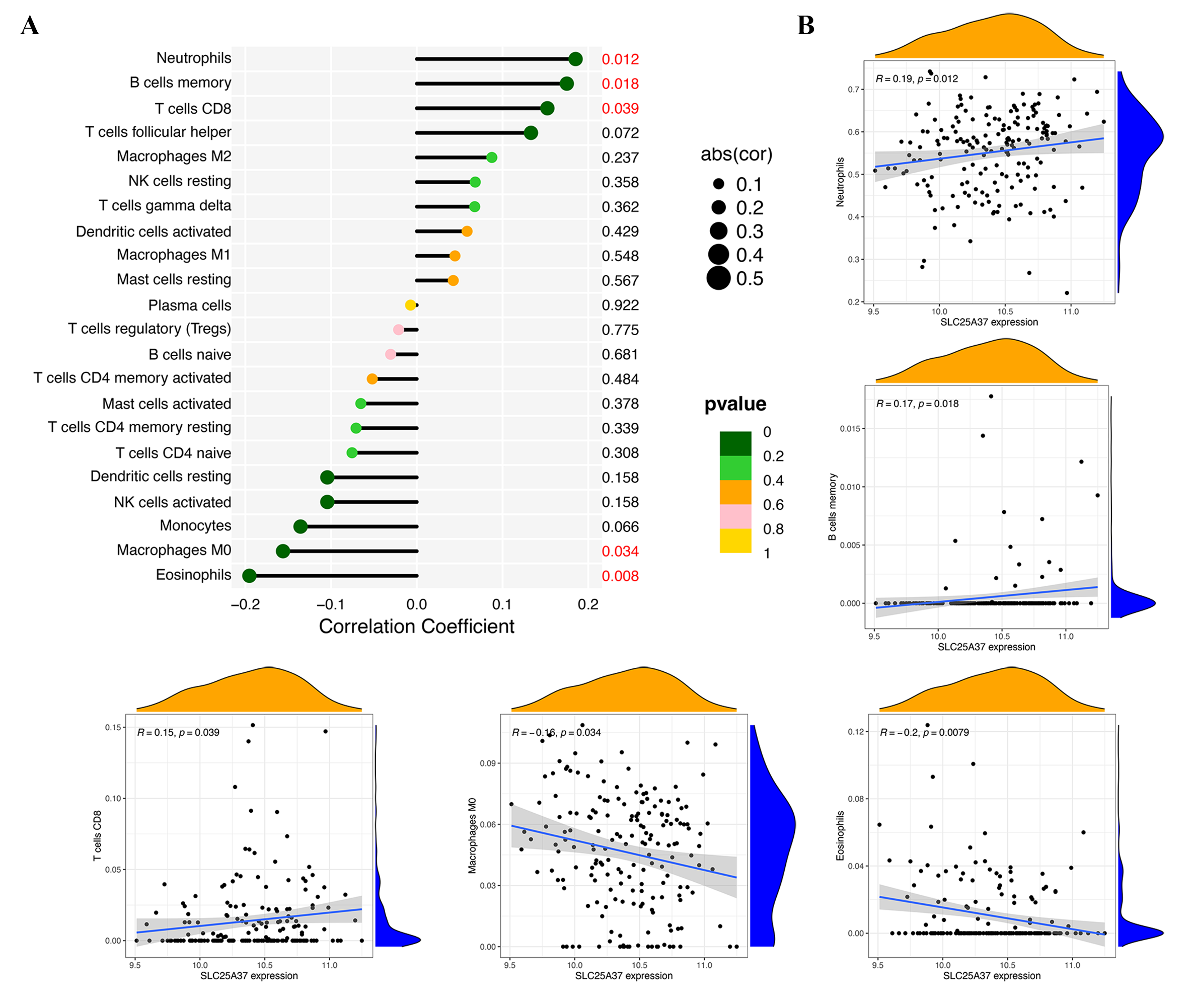

Supplement: Supplementary Figure 12 — The correlation between SLC25A17 expression and immune model. (A) Correlation Coefficient between SLC25A17 and 22 immune cell types. (B) Correlation between SLC25A17 and immune cell types with significance. [file Image_12.tif]

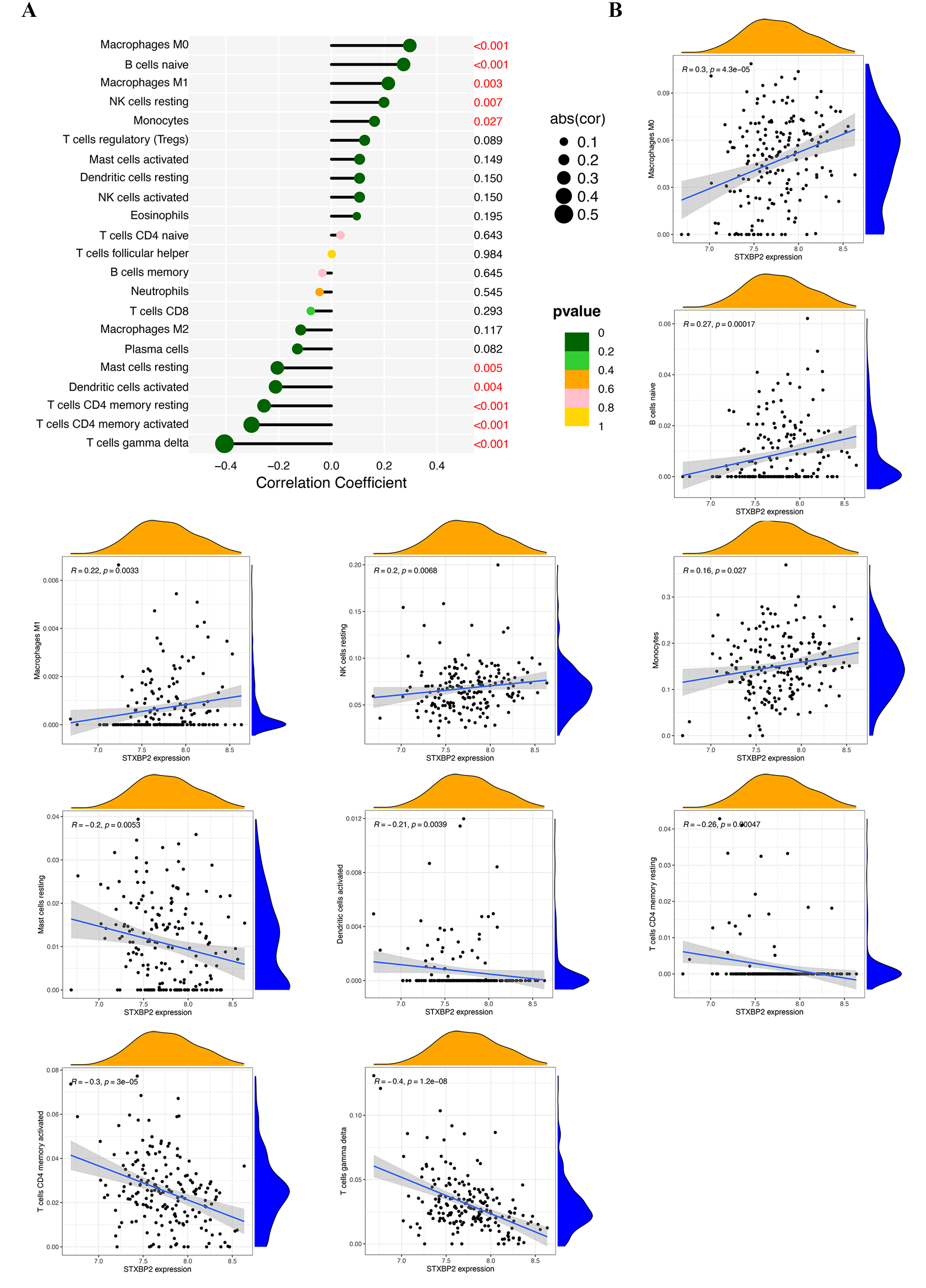

Supplement: Supplementary Figure 13 — The correlation between STXBP2 expression and immune model. (A) Correlation Coefficient between STXBP2 and 22 immune cell types. (B) Correlation between STXBP2 and immune cell types with significance. [file Image_13.tif]

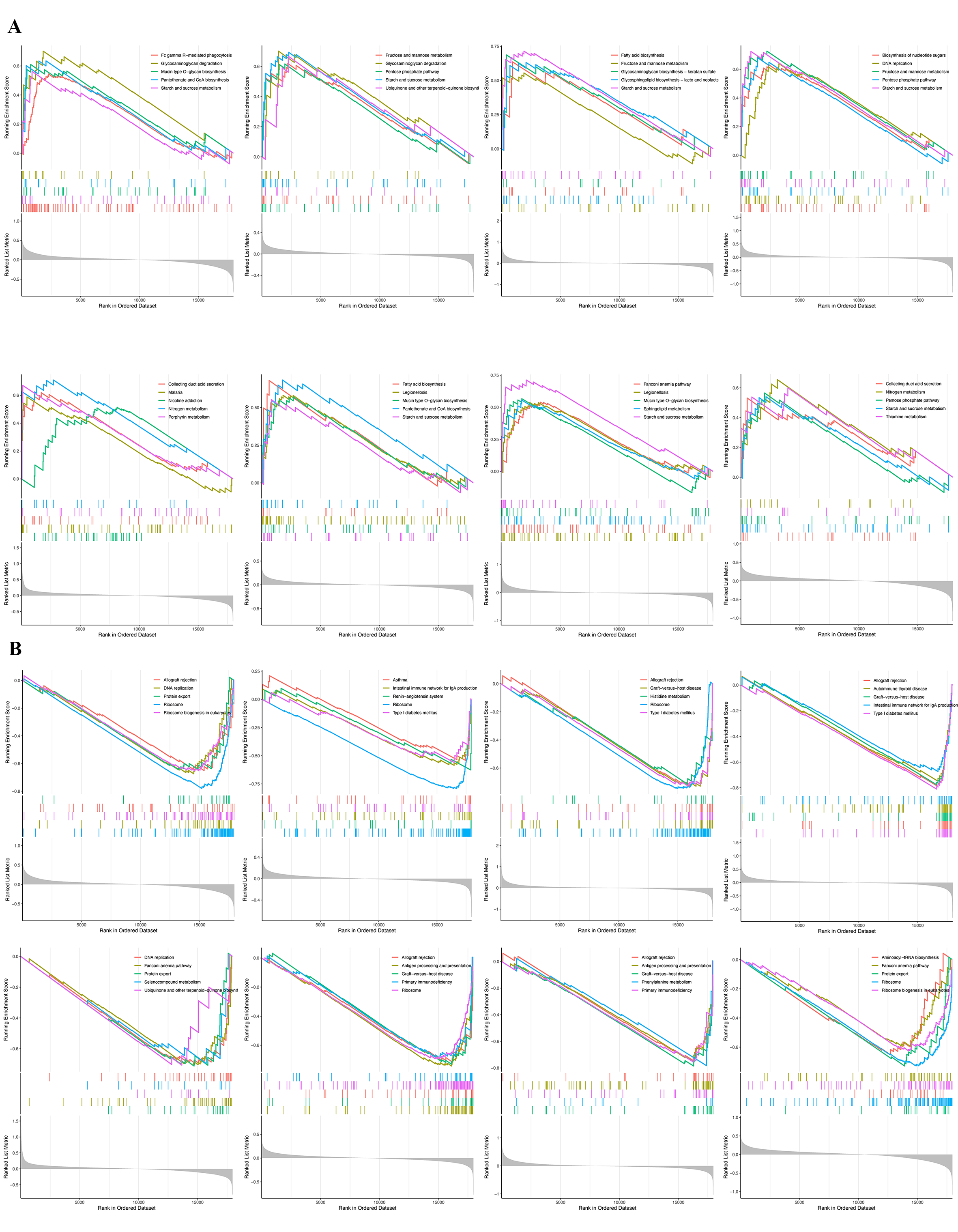

Supplement: Supplementary Figure 14 — GSEA identifies signaling pathways in the optimal hub genes. (A) The main signaling pathways that are significantly enriched under the up regulation of ALPL, ACTB, CD177, GAPDH, SLC25A37, S100A8, S100A9, and STXBP2. (B) The main signaling pathways that are significantly enriched under the downregulation of ALPL, ACTB, CD177, GAPDH, SLC25A37, S100A8, S100A9, and STXBP2. [file Image_14.tif]
