# Supplementary material for: Integrated multi-omics and artificial intelligence to explore new neutrophils clusters and potential biomarkers in sepsis with experimental validation
Source: Front Immunol. 2024 May 29;15:1377817. doi: 10.3389/fimmu.2024.1377817 (PMC11167131; doi:10.3389/fimmu.2024.1377817)
Supplement: Supplementary file 15 [file Table_1.docx]

**Supplementary Table 1**

**Information of GSE167363, GSE57065, and GSE95233**

| **GSE167363 (https://www.ncbi.nlm.nih.gov/geo/query/acc.cgi)** | |
| --- | --- |
| Status | Public on Mar 03, 2021 |
| Title | Dynamic changes in human single cell transcriptional signatures during fatal sepsis. |
| Organism | Homo sapiens |
| Experiment type | Expression profiling by high throughput sequencing. |
| Overall design | Single-cell RNA-sequencing of human peripheral blood mononuclear cells from heathy controls, survivor and non-survivor of gram-negative sepsis patients. |
| Contributor (s) | Qiu X, Li J, Bonenfant J, Jaroszewski L, Klein W, Godzik A, Nair MG |
| Web link | https://www.medrxiv.org/content/10.1101/2021.03.01.21252411v1 |
| Platforms | GPL24676 Illumina NovaSeq 6000 (Homo sapiens) |
| **GSE57065 (https://www.ncbi.nlm.nih.gov/geo/query/acc.cgi?acc=GSE57065)** | |
| Status | Public on Nov 05, 2014 |
| Title | Early and dynamic changes in gene expression in septic shock patients: a genome wide approach |
| Organism | Homo sapiens |
| Experiment type | Expression profiling by array |
| Overall design | Twenty-eight ICU patients were enrolled at the onset of septic shock. Blood samples were collected within 30 minutes, 24 and 48 hours after septic shock and compared to twenty-five healthy volunteers. |
| Contributor (s) | Cazalis MA, Lepape A, Venet F, Frager F, Mougin B, Paye M, Pachot A, Monneret G, Textoris J |
| Platforms | GPL570 [HG-U133_Plus_2] Affymetrix Human Genome U133 Plus 2.0 Array |
| **GSE95233 (https://www.ncbi.nlm.nih.gov/geo/query/acc.cgi)** | |
| Status | Public on Nov 27, 2017 |
| Title | Fractalkine receptor CX3CR1 and leukocyte Ig-like receptor B2 LILRB2 are prognostic biomarkers in septic shock |
| Organism | Homo sapiens |
| Experiment type | Expression profiling by array |
| Overall design | 51 septic shock patients and 22 healthy volunteers were included in this study. Septic shock patients were sampled twice, at admission, and a second time at D2 or D3. Admission samples from septic shock patients were compared to healthy volunteers, and according to day 28 survival status. Modulation of gene expression between the 2 time points was also analyzed according to day 28 survival. |
| Contributor (s) | Pachot A, Cazalis M, Venet F, Cerrato E, Monneret G, Lepape A, Textoris J |
| Platforms (1) | GPL570 [HG-U133_Plus_2] Affymetrix Human Genome U133 Plus 2.0 Array |
